# Extracellular DNA secreted in yeast cultures is metabolism-specific and inhibits cell proliferation

**DOI:** 10.15698/mic2023.12.810

**Published:** 2023-11-23

**Authors:** Elisabetta de Alteriis, Guido Incerti, Fabrizio Cartenì, Maria Luisa Chiusano, Chiara Colantuono, Emanuela Palomba, Pasquale Termolino, Francesco Monticolo, Alfonso Esposito, Giuliano Bonanomi, Rosanna Capparelli, Marco Iannaccone, Alessandro Foscari, Carmine Landi, Palma Parascandola, Massimo Sanchez, Valentina Tirelli, Bruna de Falco, Virginia Lanzotti, Stefano Mazzoleni

**Affiliations:** 1 Department of Biology, University of Naples “Federico II”, Via Cinthia 26, 80126 Naples, Italy.; 2 Department of Agricultural, Food, Environmental and Animal Sciences, University of Udine, via delle Scienze 206, 33100 Udine, Italy.; 3 Department of Agricultural Sciences, University of Naples “Federico II”, via Università 100, 80055 Portici (NA), Italy.; 4 Institute of Biosciences and Bioresources CNR, Via Università 133, 80055 Portici (NA), Italy.; 5 Cutaneous Biology Research Center, Massachusetts General Hospital, Boston, MA, USA.; 6 Task Force Microbiome - University of Naples “Federico II“.; 7 Laboratory of Biotechnological Processes for Energy and Industry, ENEA, Via Anguillarese, 301, - 00123 Rome, Italy.; 8 Department of Industrial Engineering, Università degli Studi di Salerno, Via Giovanni Paolo II 132, 84084 Fisciano, SA, Italy-; 9 Istituto Superiore di Sanità (ISS) Core Facilities, Viale Regina Elena 299, 00161 Rome, Italy.

**Keywords:** exDNA, eccDNA, self-DNA, 1H NMR, metabolomics, cell cycle

## Abstract

Extracellular DNA (exDNA) can be actively released by living cells and different putative functions have been attributed to it. Further, homologous exDNA has been reported to exert species-specific inhibitory effects on several organisms. Here, we demonstrate by different experimental evidence, including ^1^H-NMR metabolomic fingerprint, that the growth rate decline in *Saccharomyces cerevisiae* fed-batch cultures is determined by the accumulation of exDNA in the medium. Sequencing of such secreted exDNA represents a portion of the entire genome, showing a great similarity with extrachromosomal circular DNA (eccDNA) already reported inside yeast cells. The recovered DNA molecules were mostly single strands and specifically associated to the yeast metabolism displayed during cell growth. Flow cytometric analysis showed that the observed growth inhibition by exDNA corresponded to an arrest in the S phase of the cell cycle. These unprecedented findings open a new scenario on the functional role of exDNA produced by living cells.

## INTRODUCTION

Extracellular DNA (exDNA) has been widely reported as cell free DNA in different environments [1–5]. Moreover, exDNA may be part of extracellular structures such as the matrix of microbial biofilms [6, 7], the outer leaflet of the plasma membrane of human cells [8], and as a component of defensive structures in animals or plants, known as neutrophil [9] and root-extracellular traps, respectively [10, 11].

ExDNA is also found in biological fluids *in vivo* as well as in the culture medium of many cell types *in vitro* [10, 12–14]. The occurrence of exDNA *in vivo* has been discussed regarding the origins, structures, and different specific functions [13]. ExDNA in biofluids derives from cellular breakdown mechanisms (damaged, dying and/or dead cells), but also from active release from living cells [1, 14]. Indeed, the release of DNA by blood human cells has been described since 1976 [15] and mostly reported in sera and/or plasma of oncological patients since 2007 [16]. The exDNA can be found in molecular complexes with lipids and proteins or kept inside vesicles, so being protected from degradation by nucleases or recognition by immune cells [12, 17]. In the microbial world, many bacteria are also known to actively release DNA in a lysis-independent way, led by quorum-sensing mechanisms [18, 19]. Different roles have been attributed to exDNA delivered from bacterial cells in the environment, where it can be source of genetic material for Horizontal Gene Transfer (HGT), conferring novel functions to microbial cells, or, also, becoming a stabilizing component of the extracellular matrix in the case of biofilm producer species [20, 21].

In the context of ecological research, in studies on natural decomposition of organic matter and plant-soil negative feedback, a species-specific inhibitory effect of extracellular self-DNA was reported in plants, on both root growth and seed germination [22]. The same authors [23] observed that inhibition by self-exDNA was not limited to plants, being also revealed in experiments on different species, including bacteria, protozoa, algae, fungi, and insects, whereas no inhibitory effect was observed in all species when treated with heterologous exDNA. Also, it has been shown that in both freshwater and marine microalgae self-exDNA affected growth by arresting the cell cycle and favouring aggregates formation [24]. Moreover, the onset of developmental defects and DNA damage response to self-DNA has been reported in the model animal *Caenorhabditis elegans* [25]. Recently, in the model plant *Arabidopsis thaliana,* the detailed investigation on transcriptomic and metabolomic responses related to exposure to self-exDNA showed a generalized effect on down-regulation of gene expression [26], associated with a significant accumulation of RNA constituents, along with AMP and GMP, with their cyclic analogues and methylated forms [27].

All the inhibitory effects reported above have been experimentally observed by treatments with random fragments of genomic self-DNA, either extracted or released by decomposition of dead cells.

In the different context of yeast biomass production [28–30], a theoretical Systems Dynamics model described the aerobic fed-batch cultures of the yeast *Saccharomyces cerevisiae* [31]. The model clearly predicted the dynamics of cell density profiles, showing that the growth decline of the population could not be related to the typical end-products of yeast fermentation (ethanol and acetate), but rather to another, yet undefined, inhibitory compound [31].

We hypothesize that the inhibitory compound in yeast fed-batch cultures could be exDNA released by the cells and accumulating in the culture medium. In yeast there is a strong and well described evidence of extra-chromosomal DNA circles, reported since a long time and related to aging [32]. This finding has been confirmed and deeply studied with isolation and sequencing of these extra-chromosomal circular DNA (eccDNA) elements [33–35].

In the present work, we used *S. cerevisiae* in two different fed-batch cultures, characterized by either fermentative or respiratory metabolism according to the type of glucose feeding, to carry out the following investigations on:

i) yeast growth dynamics and metabolomic fingerprint profiling of the growth media by ^1^H nuclear magnetic resonance (^1^H NMR),

ii) recovery of exDNA from the media and assessment of its inhibitory effect on yeast growth,

iii) sequencing of the exDNA released by yeast exhibiting different metabolism,

iv) evaluation of the similarity between exDNA and eccDNA,

v) analysis of the yeast cell cycle by flow cytometry (FCM) in relation to the inhibition by exDNA.

## RESULTS

### Yeast achieves the same final biomass despite fermentative or respiratory metabolism

Two types of fed-batch cultures were used in the experiments: an “exponential feeding culture” (EFC) and a “limited feeding culture” (LFC), characterized by different glucose feeding profiles. The first ensuring full nutrient availability all over the run, while the latter based on a fine-tuning of the glucose feeding according to the effective population growth rate.

**Figure 1:**
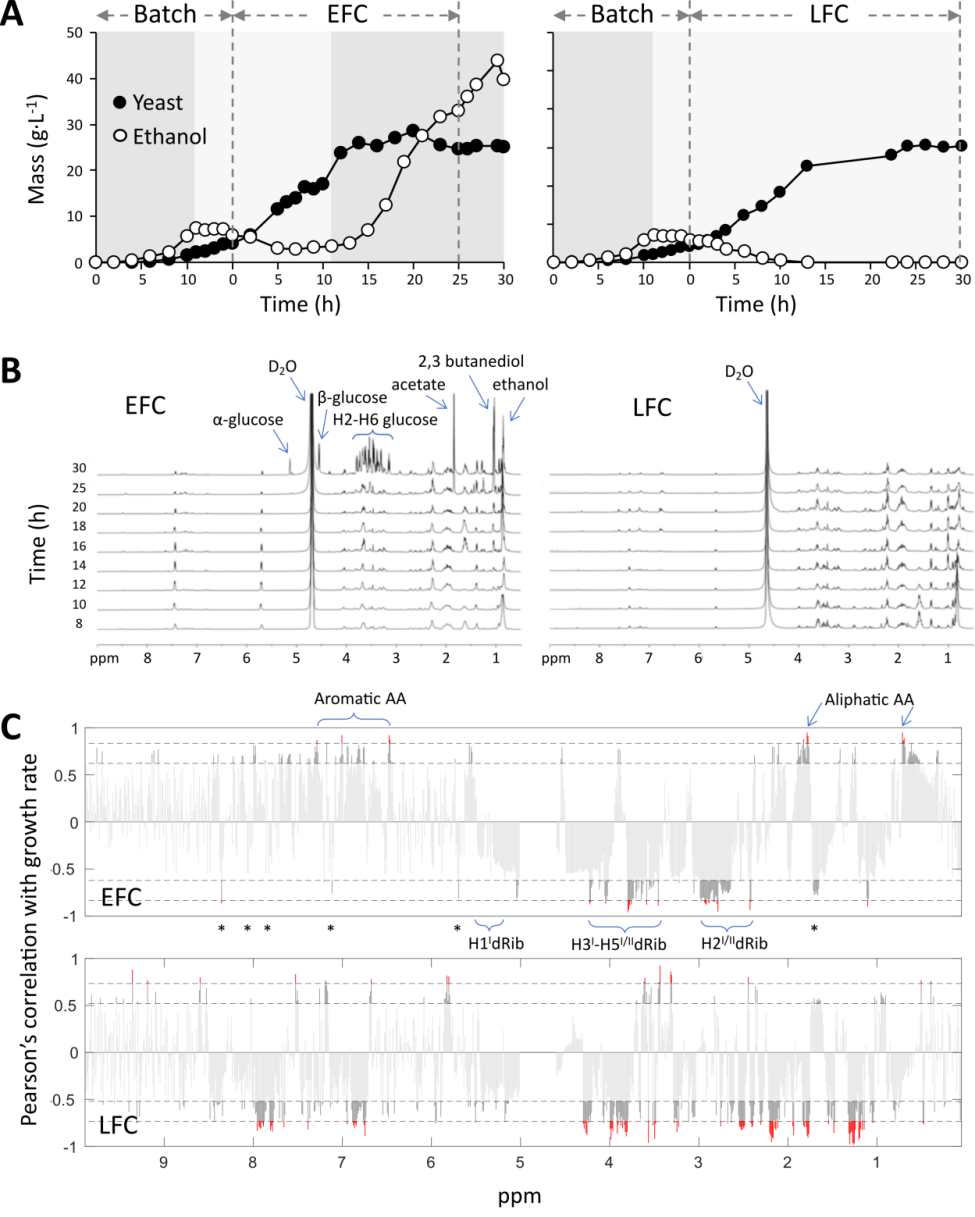
FIGURE 1: Fed-batch cultures of *S. cerevisiae* CEN.PK2-1C carried out with either exponential (EFC) or limited (LFC) nutrient feeding and associated ^1^H NMR metabolomic profiles. **(A)** Dynamic trends of yeast biomass and ethanol in the medium. Dashed vertical lines separate the batch and the feeding phases. Dark and light grey areas indicate the occurrence of either fermentative or respiratory metabolism, respectively. **(B)** ^1^H NMR metabolomic fingerprinting profiles of growth media, collected at different times between 8 and 30 h of the feeding phases. **(C)** Pearson's correlation of ^1^H NMR integrated signals of the growth media and yeast growth rates, along 30 h of the feeding phases, in conditions of either unlimited or limited nutrient availability, EFC and LFC, respectively. Labels indicate ^1^H NMR signals associated to nutrients and DNA constituents. Asterisks refer to signals diagnostic for nitrogen bases (see text for details). Red and dark grey respectively correspond to significance levels at p< 0.001 and p< 0.05. In EFC the significant negative correlations correspond to signals associated to DNA, whereas the positive correlations refer to signals associated to different nutrients. Instead, in LFC, many negative correlations still include the same DNA related signals, but also those signals linked to the limited nutrients.

Growth curves of *S. cerevisiae* in the two cultures are reported in **Fig. 1A**. The biomass increased in the initial batch phase, lasting for 15 h, with an initial typical fermentative metabolism based on glucose consumption, followed by the switch to respiratory metabolism on ethanol as substrate. During the feeding phases, both cultures achieved the same final value of biomass (about 30 g L^-1^), despite the very different feeding profiles adopted (Fig. S1A). In both cases, glucose feeding rate started at a value determining a respiratory metabolism of the yeast. In EFC, the feeding increased exponentially during the whole run (Fig. S1A), whereas in LFC, after an initial exponential increase, the feeding was progressively decreased following the biomass logistic growth (Fig. S1A). In both cultures, the growth rate declined after 8-10 h, reflecting the onset of a negative feedback on yeast proliferation. This occurred also in EFC, regardless the unlimited nutrient availability in the bioreactor (Fig. S1A). The exponential and the “limited” feeding in EFC and LFC respectively, together with the onset of the growth decline and arrest, corresponded to different amounts of residual glucose in the media, increasing in EFC, while not accumulating in LFC (Fig. S1B).

Despite the identical biomass growth in the two cultures, major differences were evident for ethanol accumulation in the vessels, which was indicative of the different occurrence of a fermentative metabolism (**Fig. 1A**). In EFC, ethanol was never completely respired and after 12 h of the feeding phase started to accumulate in the medium, indicating the shift from a respiratory to a fermentative metabolism. This reflected the uncoupling between feeding and growth rate in the culture. Differently, in LFC, the progressive decrease of feeding corresponded to the maintenance of a respiratory metabolism all over the run, with complete exhaustion of ethanol in the medium (**Fig. 1A**).

### ^1^H NMR metabolomic fingerprints of culture media reflect the feeding conditions

^1^H NMR spectra acquired for supernatants from EFC and LFC, collected at different times of the feeding phase are reported in **Fig. 1B**. They represent the metabolomic fingerprinting profiles of the two cultures (EFC and LFC) during the feeding phase. The ^1^H NMR spectrum of the nutri-ent medium filling the bioreactor before inoculation is also reported (Fig. S2). In this profile the key signals of glucose were well distinguishable: a broad singlet at δ 5.20 for H-1 of α-glucose and the doublet, J=7Hz, at δ 4.61 for H-1 of β-glucose. The remaining glucose signals (H2-H6) resonated in the δ 3.15-3.90 interval. Moreover, other relevant compounds of the nutritional medium were uracil (two coupled doublets at δ 5.75 and δ 7.49), tryptophane (multiplets at δ 7.20-7.40), leucine (doublet at δ 1.00), glutamic acid (multiplets at δ 2.05 and 2.25), histidine (multiplet a δ 2.98).

The metabolomic fingerprinting profile of EFC during the feeding phase (**Fig. 1B**; from 8 to 30 h) clearly shows that the glucose signals initially disappeared (compared to the initial starting medium, in Fig. S2), as expected when the feeding rate equalled the population growth rate and consequently no residual glucose was present in the medium. Instead, the signals of other nutrients remained evident during the run, thus indicating that no nutrient limitation occurred during the cultivation.

The time series of the EFC showed the progressive increase of ethanol, identified by a triplet at δ 0.92, increasing after the appearance of growth decline followed by the shift towards fermentation. Also, other two by-products of glucose fermentation were detected at a late stage: acetate and 2-3 butanediol with singlet signals at δ 1.90 and 1.10, respectively. A late increase of unconsumed glucose is shown by the signals in the carbohydrate region: δ 3.15-3.90 (H2-H_2_6), δ 5.20 H1 α-glucose and δ 4.60 H1 β-glucose.

In the spectra of LFC medium **(Fig. 1B)**, the signals for nutrients were like to those reported above for EFC, whereas the signals of ethanol and other fermentative by-products were absent, reflecting the full respiratory metabolism maintained by yeast during the whole LFC run.

A further analysis of the spectra at higher intensity (Fig. S3) showed the progressive appearance of some signals in the aromatic and carbohydrate regions. Particularly evident are the signals in the 3.40-4-40 ppm interval, increasing during the runs, diagnostic for H3^I^/H4^I^/H5^I^/H5^II^ of deoxyribose and also in the range 2.30-2.80 ppm, indicative for H2^I^/H2^II^. Moreover, with lower intensity, the signals for different nitrogen bases were observed at δ 8.30-8.40 (H-8, dA), 8.10-8.20 (H-2, dA), 7.85-7.95 (H-8, dG), 7.70-7.85 (H-6, dC), and 1.75-1.85 (Me, dT) (Fig. S3).

### DNA-related signals in ^1^H NMR spectra show significant negative correlation with yeast growth rates

The Pearson's correlation of ^1^H NMR signals with yeast growth rate during the feeding phases (**Fig. 1C**), in the case of EFC highlighted a significant positive correlation for signals of aliphatic amino acids at δ 0.60-0.75 and δ 1.80-2.20, and of aromatic amino acids between δ 6.50 and 7.30. Differently, very significant negative correlations (p<0.001) were found in the spectra for signals compatible with the pentose deoxyribose. Thus, in the carbohydrate region signals in the δ 3.40-4.40 interval were attributed to H-3^I^/H-4^I^/H-5^I^/H-5^II^, while additional signals at δ 2.30-2.80 were ascribable to H-2^I^/H-2^II^ (**Fig. 1C**). Moreover, a negative correlation was observed for signals at δ 5.60-6.30 related to the H-1' anomeric protons of the deoxyribose units (**Fig. 1C**). Interestingly, negative correlations were also observed for the set of signals related to aromatic heterocyclic nitrogen bases mentioned above in the spectra analysis, although in some cases with border line statistical significance. They were: δ 8.30-8.40 (H-8, dA), 8.10-8.20 (H-2, dA), 7.85-7.95 (H-8, dG), 7.70-7.85 (H-6, dC), 7.15-7.30 (H-6, dT), 5.90-6.05 (H-5, dC) and 1.75-1.85 (Me, dT) (**Fig. 1C**).

These values agreed with those of standard nucleotides available in our laboratory and with data reported in the literature[36, 37]. Of note, the different level of significance between deoxyribose (all signals at p < 0.001) and nitrogen bases (most signals at p < 0.05) reflects their relative abundance in the DNA structure.

Looking at the correlation analysis of LFC spectra, a very different pattern appeared with reduced significant positive correlations and stronger reversed correlation for the aliphatic amino acid signals, showing negative correlations at δ 2.10, 1,70 and 1.25. This opposite correlation to that observed in the case of EFC, was associated to the progressively limited feeding, which apparently set the occurrence of a reduced nutrient availability condition. Interestingly, the signals associated to both deoxyribose and nitrogen bases of DNA still showed the same negative correlation found for EFC (**Fig. 1C**).

### ExDNA accumulating in the culture media produces an inhibitory effect on yeast growth

**Figure 2:**
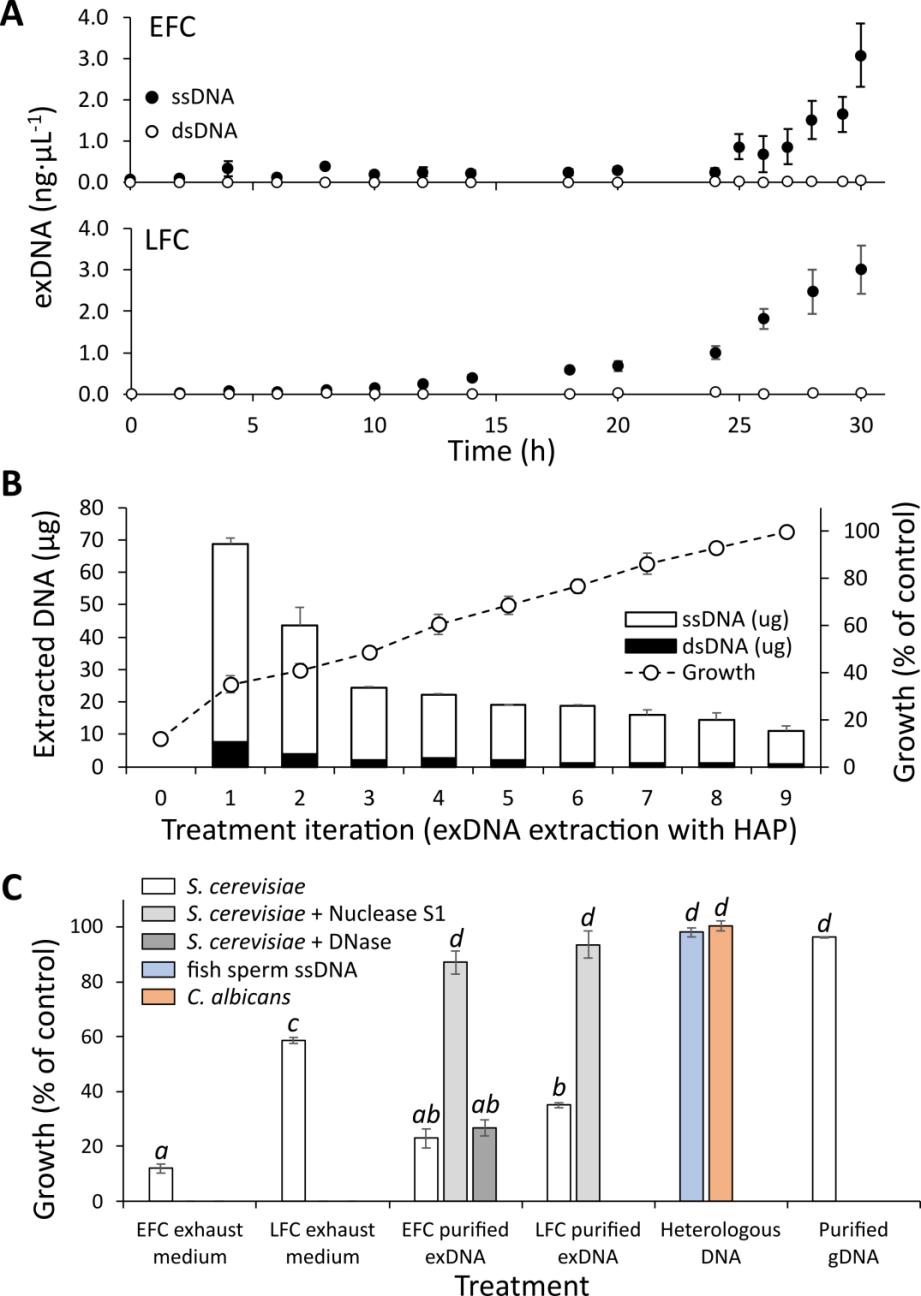
FIGURE 2: ExDNA accumulates in the medium and produces an inhibitory effect on yeast growth. **(A)** Quantification of single and double stranded exDNA in EFC and LFC media, during the feeding phases. **(B)** Stacked bars represent the amounts of single (white) and double (black) stranded exDNA purified from the exhausted medium of EFC, by nine subsequent extractions with HAP. Circles refer to yeast growth in batch cultures with addition of the eluate obtained by each HAP extraction. Cell growth was determined by A_590_ and expressed as percentage of the control. A clear reduction of the inhibitory effect of the eluates is observed with a complete recovery of 100% growth after nine HAP treatments. **(C)** Effects of exhausted media, exDNA purified from EFC and LFC supernatants and heterologous DNA on yeast growth in batch cultures. Growth was determined by A_590_ after 12 h incubation expressed as percentage of the control. Data refer to mean and standard deviation of 3 replicates. Different letters above bars indicate statistically significant pairwise differences (Tuckey post-hoc test after one-way ANOVA for the effect of treatments on yeast growth). The highest inhibition level is observed for exDNA from EFC medium, whereas no significant inhibitory effect is produced by heterologous DNAs (fish sperm ssDNA, or genomic DNA of *C. albicans*) and genomic DNA of *S.cerevisiae* CEN.PK2-1C. A significant recovery of growth was observed when exDNA was pretreated with S1 nuclease, whereas no recovery was found using DNAse.

An increasing amount of exDNA in the medium of both EFC and LFC along the feeding phase (**Fig. 2A**) was observed. The final recovered amounts of exDNA were quantitatively comparable for EFC and LFC, with a final value of about 3 ng µL^-1^ in both media. In all the same samples, no quantities of RNA were found to be detectable. Noteworthy, the purified exDNA was dominated by single strand fragments always corresponding to more than 98% of the total extracted DNA (**Fig. 2A**). Further evidence of the occurrence of exDNA in the media is shown by direct amplification of EFC and LFC supernatants (Fig. S4).

Experiments in *de novo* batch cultures were performed to verify the inhibitory effect on yeast growth of the exhausted media from EFC and LFC. The exhausted culture medium added to a fresh substrate of a batch culture determined a strong inhibition of yeast growth. The iterated removal of exDNA from the exhausted medium of EFC by the HAP adsorption procedure corresponded to a progressive reduction of such inhibitory effect, starting from a growth of only 12% of the control and recovering, after nine extraction steps, the full growth performance (**Fig. 2B**). Noteworthy, the HAP adsorption procedure confirmed the findings reported in **Fig. 2A**, allowing to recover, also in this case, a large dominance of single strand fragments, with double strands always less than 2% of the total extracted amount (**Fig. 2B**). On the same samples, no detectable quantities of RNA were observed.

So, a clear growth inhibition could be observed when the exhausted media from EFC and LFC were added to *de novo* batch cultures (**Fig. 2C)**. The inhibitory effect was significantly higher with the EFC medium compared to the LFC one (**Fig. 2C)**.

An even higher and very significant inhibitory effect was observed with the direct addition of the purified exDNA from both EFC and LFC to the batch cultures (**Fig. 2C**). Again, also in this case as for the exhausted media, the inhibition effect was greater with exDNA extracted from EFC compared to LFC. In contrast, no inhibition was observed in the treatment with heterologous DNA, neither the ssDNA of fish sperm, nor the genomic DNA from *C. albicans* and *S. cerevisiae*, both even at 30 ng µL^-1^. A significant recovery of growth was observed when exDNA was pretreated with S1 nuclease, whereas no recovery was observed using DNAse (**Fig. 2C).**

Another experiment is reported in Fig. S5 demonstrating the inhibitory effect on yeast growth by DNA obtained by the direct amplification of the EFC supernatant. This effect of dsDNA was found to occur at significantly higher concentrations (40 and 80 ng µL^-1^) than in the case of the extracted ssDNA (3 ng µL^-1^) reported in **Fig. 2C**. Also in this case, the inhibitory effect was absent in the presence of different heterologous DNAs, as well as after pre-treatment of the amplified DNA with DNase (Fig. S5).

Moreover, an experiment with RNA extracted from the *S. cerevisiae* CEN.PK2-1C strain and added to the culture medium, in parallel with heterologous RNA, showed no inhibitory effects on yeast growth (Table S1).

### ExDNA is only a portion of the entire genome with specific differences associated to active metabolism

The DNA fragments recovered from EFC and LFC exhausted media collected at the end of the runs, where yeast displayed either fermentative or respiratory metabolism, respectively, were analyzed by high-throughput sequencing and bioinformatics (detailed results are in Supplemental Dataset S1). The mapping procedure, showed 2133 and 12030 contigs for EFC and LFC, respectively, aligning on the yeast genome (**Table 1**). Such difference between the samples was consistent when also considering the total length in kbp of the contigs coverage (**Table 1**). Additionally, DNA fragments were also obtained from EFC medium during the exponential growth phase (EFC-6 h), when metabolism was still respiratory, and submitted to the same bioinformatic pipeline, producing a total of 3885 contigs (**Table 1**).

**Table 1.** Descriptive metrics of the contigs resulting from the high-throughput sequencing of exDNA fragments purified from the culture media samples. For each sample, the number, length and mean FPKM values are shown for all contigs, and separately for contigs either shorter or longer than 100 bp and, among the latter, for different classes of FPKM ranges: > 1000, 100-to-1000, 10-to-100, < 10.

	**Sample**		
	**EFC**	**EFC_6h**	**LFC**
**Contigs > 100 bp**			
*FPKM > 1000*			
N	10	7	2
Total length (kpb)	32	15	19
Mean FPKM	2,620	3,818	3,381
*100 < FPKM < 1000*			
N	57	35	3
Total length (kpb)	160	175	59
Mean FPKM	326	254	136
*10 < FPKM < 100*			
N	1,469	981	90
Total length (kpb)	471	795	957
Mean FPKM	19	18	23
*FPKM < 10*			
N	20	2,233	11,928
Total length (kpb)	3	454	3,729
Mean FPKM	10	6	2
*All FPKM*			
N	1,556	3,256	12,023
Mean length (bp)	428	442	396
Total length (kpb)	665	1,438	4,763
Mean FPKM	47	21	3
**Contigs < 100 bp**			
*All FPKM*			
N	577	629	7
Mean length (bp)	26	27	99
Total length (kpb)	15	17	1
Mean FPKM	123	69	2
**All contigs**			
N	2,133	3,885	12,030
Mean length (bp)	319	374	396
Total length (kpb)	680	1,455	4,763
Mean FPKM	68	29	3

Contigs were relatively evenly distributed across all chromosomes in all samples, with contigs counts per Mbp across chromosomes equal to 165 ± 35, 313 ± 40 and 974 ± 110 in EFC, EFC-6h and LFC exhausted media, respectively. A remarkable exception to such pattern was the distribution of contigs in mitochondrial DNA. Indeed, opposite to the nuclear chromosomes, mitochondrial DNA was far more represented in EFC (1251 contigs per Mbp, a value of almost one order of magnitude higher than that of nuclear DNA) and in EFC-6h (1228 contigs per Mbp) as compared to LFC (374 contigs per Mbp, a value much lower than that of nuclear DNA) exhausted medium.

Fragment length was highly variable, but with similar mean values in both media (319, 374 and 396 bp in EFC, EFC-6h and LFC, respectively, **Table 1**), ranging between 11 bp and 14.3 kbp in EFC, between 11 bp and 16.8 kb in EFC-6h, and between 98 bp and 46.9 kbp in LFC. However, counts of contigs shorter than 100 bp were 577 in EFC, 629 in EFC-6h, and only seven in LFC (**Table 1**). Then, after filtering the contigs for length > 100 bp, reads counts were also highly variable, showing different distribution patterns in the sequenced samples (**Table 1**). Among the ten most abundant sequences of length > 100 bp and FPKM (fragments per kilobase per million) > 1000 (**Table 1**), two map on chromosome VII and two more on chromosome XVI, one on each of the chromosomes IV, XII and XIII, and three map on the mitochondrial chromosome. It is worth noting that the latter mitochondrial fragments are far shorter than all the others. Differently, in LFC we found a homogeneous reads distribution with only two contigs with FPKM values higher than 1000 (**Table 1**), both mapping on chromosome XII. Finally, the contigs resulting from EFC-6h showed an intermediate reads distribution pattern, as compared to the other exhausted media (**Table 1**). Only seven contigs showed FPKM > 1000, one on each of the chromosomes III, IV, X, XII, XIII and XIV, and one on the mitochondrial chromosome. Interestingly, the most abundant contigs mapping on the chromosome XII in all the three samples fall in the region including ribosomal DNA (Supplemental Dataset S1).

Considering all the DNA fragments recovered from the exhausted media, their sequences correspond overall to 5.6% (0.680 Mbp) and 39.2% (4.763 Mbp) of the whole yeast genome, for EFC and LFC, respectively. The same figure for the EFC-6h is 12.0% (1.455 Mbp).

**Figure 3:**
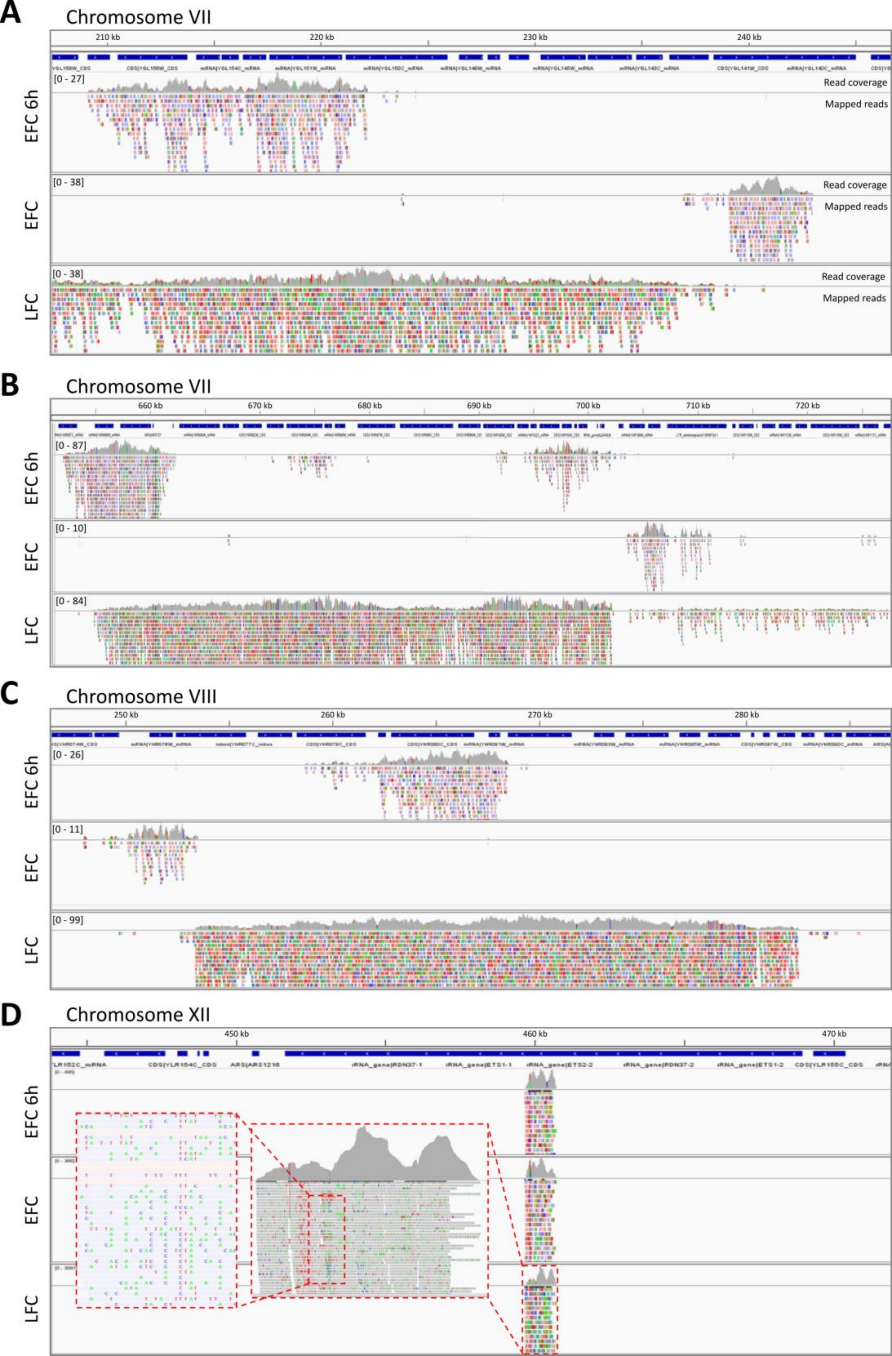
FIGURE 3: ExDNA sequencing from yeast culture media reveals specific differences associated to active metabolism. Examples of nucleotide reads mapped to the S288C *S. cerevisiae* reference genome. Shown are samples of sequenced exDNA collected from media of early respiratory EFC (EFC-6h), late fermentative EFC (EFC), and respiratory LFC (LFC). The reads are aligned on the corresponding parts of genomic regions of different chromosomes: chromosome VII **(A, B)**, chromosome VIII **(C)**, and chromosome XII **(D)**. Blue bars refer to specific sequence features of the mapped regions. Grey histograms represent mapped reads coverage. Coloured boxes indicate individual reads nucleotide mismatches, with respect to the reference genome sequences. Magnification of a portion of chromosomes XII, showing the high degree of mismatch rate, is shown in the inset (D).

By a comparative analysis of the sequencing results of the exDNA purified from EFC, EFC-6h and LFC along the different genomic regions and the gene loci, a striking pattern was observed (**Fig. 3**). Indeed, in many cases, the reads of the exDNA from EFC and LFC mapped on not overlapping genomic regions, as in the examples reported for chromosome VII and VIII. However, noteworthy, the set of sequence reads recovered from the EFC-6h supernatant appears to map in regions overlapping and mostly included in those associated to LFC. Instead, there were not overlapping genome regions between the reads at EFC at 6h and those from the EFC exhausted medium (**Fig. 3**). Other examples of differences between EFC and LFC are reported for chromosomes XI and XIV with regions exclusive for LFC or chromosome IV and VII for EFC (Fig. S6). Instead, noteworthy, a very different pattern was observed for chromosome XII regions associated to rDNA that were found overlapping in all media, both when considering the unique mapped reads (**Fig. 3**) and the multiple mapping ones (Fig. S6A)

It is evident that such different loci coverage could be associated to either fermentative or respiratory metabolism according to culture conditions. Based on KEGG database, an analysis of the genes number exclusively associated to respiratory metabolism (TCA cycle, pentose phosphate pathway, and oxidative phosphorylation) or common to both respiration and fermentation (genes of glycolysis/gluconeogenesis and cell cycle) was done in the sequenced exDNA fragments, recovered from the exhausted media of EFC, LFC, and EFC-6 h and reported in **Table 2**.

**Table 2.** Number and percentage of genes in sequenced exDNA from exhausted media of different fedbatch conditions (EFC and LFC at the end of the runs and EFC at 6h) compared to eccDNA [33]. The percentages refer to genes included in specific KEGG metabolic pathways either exclusively associated to respiratory metabolism (TCA cycle, pentose phosphate, oxidative phosphorylation) or common to both fermentative and respiratory metabolism (glucose pathways and cell cycle).

	**Genes in exDNA**	**TCA cycle**		**Pentose phosphate**		**Oxidative phosphorylation**		**Glycolysis / gluconeogenesis**		**Cell cycle**	
**Genes in KEGG pathway**		31		28		76		55		130	
**EFC**	1001	2	6%	3	11%	0	0%	10	18%	24	18%
**EFC_6h**	1823	7	23%	11	39%	2	3%	17	31%	45	35%
**LFC**	4632	22	71%	23	82%	4	5%	40	73%	101	78%
**eccDNA**	1861	16	52%	10	36%	2	3%	0	0%	42	32%

Data in **Table 2** show a comparison among the sequences recovered from the different supernatants, reporting the number and percentage of genes in the different growth conditions. Focusing on the coding regions representative of the metabolic processes, clear differences among the samples are evident. In EFC, it is apparent the reduced number of genes related to respiratory metabolism compared to LFC and EFC-6 h, the latter both exhibiting a respiratory metabolism. The same analysis, performed for the eccDNA recovered from 48 h respiratory cells [33], shows a similarity of such eccDNA with the exDNA from LFC and EFC-6h. Instead, constitutive genes related to central glucose pathways and cell cycle were not significantly different among the samples from different origins (**Table 2**).

Moreover, the reads of sequenced exDNA (**Fig. 3**), showed a high, randomly distributed sequence variability, with many nucleotide mismatches as compared to the corresponding sequences in the reference genome. Further investigation confirmed their specific alignment of exDNA reads on *S. cerevisiae* genome and their specific variants. First, by a BLAST alignment of three different reads mapping on the rDNA region against the nucleotide database (Fig. S7), exclusive hits were found on the same corresponding regions of *S. cerevisiae*, i.e., on rDNA on chromosome XII. Second, repeating the BLAST alignment, after excluding *S. cerevisiae* records from the nucleotide database, produced very few hits, with low similarity values (Fig. S8).

### ExDNA sequences correspond to eccDNA, but depending on active metabolism

Comparing exDNA fragments with extrachromosomal DNA circles accumulated in yeast cells grown for 48 h under respiratory metabolism conditions (eccDNA) [33], we found a striking correspondence between LFC exDNA and eccDNA. They showed similarity ratio of 83.6%, when calculated as the number of overlapping fragments (i.e., contigs of exDNA mapping on regions of the reference genome overlapping those where eccDNA contigs map) on the total number of eccDNA fragments, and of 91.2% when calculated as the length in bp of overlapping fragments on the total eccDNA length (Supplemental Dataset S1). However, it should be noted that exDNA included many fragments in addition to those overlapping eccDNA. Differently, exDNA from EFC exhausted medium had much less in common with eccDNA, with a similarity ratio of 20% based on overlapping fragments (39.5% in bp), while exDNA from EFC-6h, still under respiratory metabolism, showed higher similarity to eccDNA (similarity ratios of 27.8% and 45.1%, based on overlapping fragments and bp, respectively), closer to the values reported for LFC (Supplemental Dataset S1).

**Figure 4:**
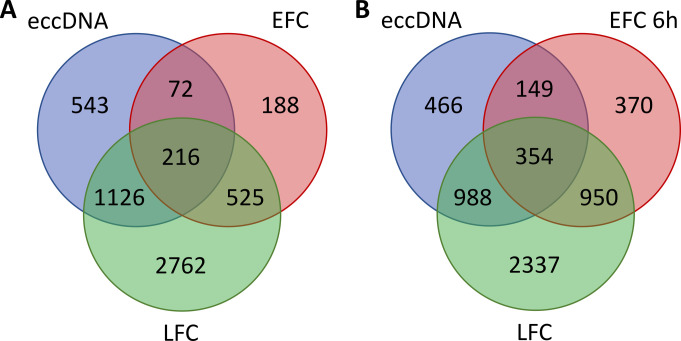
FIGURE 4: ExDNA sequences correspond to subsets of eccDNA depending on active metabolism. Venn diagrams comparing genes found in eccDNA with those occurring in exDNA from different growth media. Genes completely or partially represented in at least one *S. cerevisiae* eccDNA sample (N=1957) [33], are compared to those occurring in exDNA purified from exhausted media of LFC and either EFC (A) or EFC 6h (B). ExDNA from respiratory LFC and, to a lesser extent EFC 6h, shows higher similarity with eccDNA as compared to the fermentative EFC.

When the comparisons were limited to coding sequences (**Fig. 4** and Supplemental Dataset S2), we found that 1342 (216 + 1126, **Fig. 4A**) eccDNA genes, correspond-ing to 70% of all the 1957 eccDNA genes, were also represented in LFC exDNA fragments. Differently, only 288 (216 + 72) eccDNA genes, less than 16% of all the eccDNA genes, were represented in EFC exDNA fragments (**Fig. 4A**). Interestingly, when considering the coding sequences in DNA fragments purified from the EFC-6h medium (**Fig. 4B**; still respiratory condition), we found a higher similarity with both eccDNA and LFC exDNA. In particular, the genes in common between EFC-6h and eccDNA were 503 (354 + 149, **Fig. 4B**), corresponding to 25.7% of all the eccDNA genes, whereas the genes in common with LFC were 1304 (354+950, **Fig. 4B**), corresponding to 28,2% of all LFC genes.

Confirming the higher similarity of eccDNA with LFC and the lower one with EFC, we found the following correspondences for the regions overlapping with high copy eccDNA: *HXT6-7* in LFC whereas in EFC only *HXT7*; *ENA* and *CUP1* only in LFC; some coding regions within the telomeric Y prime in all conditions, for example the genes *YBL113C* and *YFL066C* were found in LFC but not in EFC (Supplemental Dataset S2).

Regarding mitochondrial DNA in eccDNA, it was reported as a single fragment of 85 kbp [33], whereas the contigs of mitochondrial DNA resulting from our study in all conditions were shorter by two orders of magnitude, ranging on average between 172 and 333 bp (Supplemental Dataset S1).

Then, the overall comparisons of the exDNA purified from our yeast culture media with eccDNA clearly indicates a greater similarity of DNA from cells displaying the same metabolism. This result agrees with the outcome of KEGG pathways analysis of genes related to respiratory metabolism reported in **Table 2**.

### The onset of growth inhibition arrests the cell-cycle in S phase

**Figure 5:**
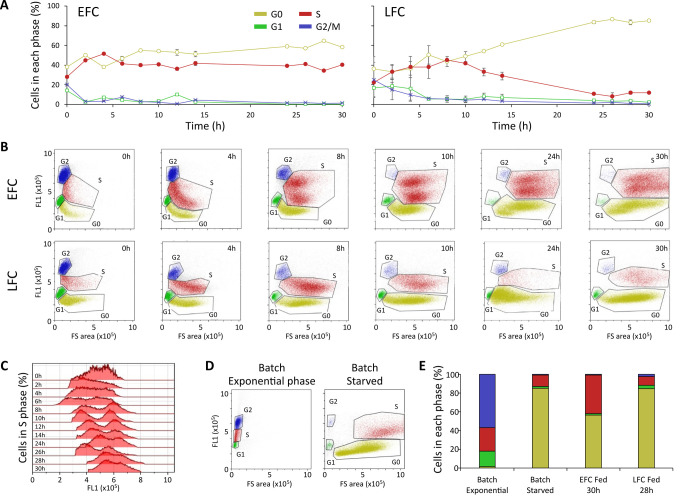
FIGURE 5: Flow cytometric analysis (FCM) of the cell cycle shows an arrest in S phase despite unlimiting nutrient availability. **(A)** Dynamic trends of the yeast population in the different cell cycle phases (G1, G0, S, G2/M) during the feeding phase of either unlimiting (EFC) or limiting (LFC) nutrient availability. **(B)** Representative outputs of the bi-dimensional FCM analyses at different times of the feeding phase of EFC and LFC. In the dot plots of each panel, G0, G1, S, and G2/M cell cycle stages are identified according to both forward scatter signal (FSC-A) and green fluorescence (FL1-A), representing cell size and DNA content per cell, respectively. **(C)** S phase heterogeneity in the yeast population during the EFC feeding phase. The histograms show the fluorescence (FL1-A) distribution during the S phase of the EFC. Starting from 8 h, a doubling of the population, in terms of fluorescence intensity, is evident, while at the end of the cultivation run a convergence towards only one fluorescence peak is observed. **(D)** Outputs of the bi-dimensional FCM analyses for a yeast population in a batch exponential and starved culture. **(E)** Comparison of the percentages of the yeast population in each phase of the cell cycle (G1, G0, S, G2/M) in different culture conditions: batch (exponential), batch (starved), EFC (30 h of feeding), LFC (30 h of feeding).

The progression of cell cycle in the yeast populations in both EFC and LFC was monitored by flow cytometry (FCM) based on the differences in DNA content and cellular size, so permitting the identification of the pre-replicative (G0 and G1), DNA synthesis (S), post-replicative and mitotic (G2/M) phases. The dynamic trends for each stage of the cell cycle in the yeast population, during both the feeding phases of EFC and LFC, are reported in **Fig. 5A**, with the corresponding outputs of the bi-dimensional FCM analyses in **Fig. 5B**.

Looking at the dynamic trends along the runs, cells were initially in active division with higher levels of G1 and G2/M states, which later declined with the progression of the cultures, whereas levels of both G0 and S cells increased, reaching values over 40%. From 10 h onwards, the two cultures showed different trends, with G0 and S states stabilizing in EFC at high comparable levels, whereas, in LFC, the cells in the S phase progressively decreased reaching a final G0 dominance (**Fig. 5A**).

In bi-dimensional FCM plots (**Fig. 5B**), it can be noticed that in EFC the population of S phase cells showed a split in two sub-sets, which was evident from 8 h onward, tending to merge again at the end of the run. Differently, such division is not observed in LFC, with only one population of cells in S phase, decreasing abundance after 10 h until the end of the run.

The **Fig. 5C** highlights the distribution of the population in the S phase during the EFC run. The occurrence of a bimodal distribution is set between 8 and 24 h, with an accumulation of a sub-population in the region with lower fluorescence intensity (early stages of S phase).

Moreover, for reference, an assessment of the cell cycle of a yeast population in a typical batch cultivation on glucose is shown, during either exponential or stationary phase, with the former being dominated by active cell division, while the latter characterized by quiescence induced by starvation, with dominance of G0 cells (**Fig. 5D**).

In **Fig. 5E**, a summary comparison of the yeast populations in the different cell cycle phases, in either batch or fed-batch cultivations, is reported. As compared to the starved batch culture, dominated by a large percentage of G0 cells, in EFC at 30 h the growth arrest corresponds to an unexpected high percentage of the population in S phase, along with the G0 cells. LFC population at 30 h, instead, shows a level of S cells comparable to the batch starved condition (**Fig. 5E**).

## DISCUSSION

In batch cultures, microbial populations exponentially proliferate, until unavoidable stationary phase is reached, due to nutrient depletion [38]. Otherwise, fed-batch cultivations, with continuous flow of nutrients to the vessel, allow prolonged growth with the achievement of higher cell densities than in batch [39–41]. However, also in such systems, the population growth always shows a logistic trend, inevitably reaching a limit to the maximal attainable cell density, despite the maintenance of nutrient availability [42–44].

In *S. cerevisiae* fed-batch cultures, the occurrence of growth curve inflections was thoroughly discussed in terms of a negative feedback due to the inhibitory effect of a growth-linked by-product accumulation [31]. The experiments presented here confirmed the occurrence of a growth rate decline in the yeast cultures under two different feeding conditions, EFC and LFC. Both cultures reached the same value of final biomass, thus reflecting a comparable occurrence of inhibition, despite the different cell metabolism displayed. Indeed, both systems started with the same exponential glucose feeding that was set in such a way to ensure a respiratory metabolism after the batch phase completion. Instead, during the EFC run, the progressive increase of feeding induced the metabolic shift to fermentation, typical for the glucose-sensitive yeast *S. cerevisiae* under high glucose concentrations [45, 46]. Differently, in LFC, the modulation of glucose feeding, with its controlled progressive reduction following the growth rate decline, avoided sugar accumulation in the medium, allowing yeast metabolism to remain respiratory for the whole run. So, the two cultivation strategies allowed us to collect and process medium samples deriving from cultures under either initial respiratory and then fermentative metabolism (early and late phases of EFC culture, respectively) or always maintained under respiratory conditions (LFC). These different metabolic dynamics are clearly displayed by the changes of ^1^H NMR profiles during the fed-batch runs.

In a previous modelling work [31] it was hypothesized that the accumulation of an inhibitory compound in yeast fed-batch cultures was proportional to cell population growth. Here, we provide evidence that the molecule inhibiting yeast proliferation is the exDNA accumulated in the medium.

The general metabolomic ^1^H NMR analysis of the culture media highlighted the statistical significance of emergent negative correlation between DNA-related ^1^H NMR signals and yeast growth rate. This result clearly supports the hypothesis of DNA as the major growth inhibitor, and it is unequivocally highlighted in EFC, with the absence of other significant negative signals for different organic compounds. Instead, in LFC, the same correlation analysis, while confirming the negative correlation of DNA signals, also showed many other nutrient related signals simultaneously associated to the growth rate decrease, as expected, because of the progressively limited feeding in this culture system. No significant increase has been observed for the ^1^H NMR signals of known putative inhibitory metabolites belonging to the classes of aromatic alcohols (2-phenylethanol, tryptophol and tyrosol) and saturated medium-chain C6, C8 and C10 fatty acids [47, 48].

Indeed, the ssDNA recovered from the exhausted media of both EFC and LFC showed accumulation profiles during the runs. Noteworthy, such exDNA increasing concentration still occurred even after reaching the growth plateau, thus reflecting a process related to a general metabolic activity rather than to cell division only. The amounts of exDNA in the media at the end of the culture runs were in the range of concentrations of exDNA reported in the media of different microbial isolates [49]. They were similar in both EFC and LFC, with ssDNA always more than 98% of the extracted amount.

The occurrence of exDNA has not been reported in *S. cerevisiae* cultures so far. Of note, however, exDNA has been detected in the exopolymeric matrix of biofilms of another yeast species, *Candida albicans*, constituting around 5% of the weight of the matrix and composed largely of random non-coding sequences [6].

Looking at the sequences of the exDNA recovered from the yeast culture media, we found a large dominance of rDNA in all the samples, i.e., of fragments associated to the ribosomal region on chromosome XII. These were found identical for both EFC and LFC, as well as in both EFC sampling times. Instead, striking differences were found between other regions associated to exDNA fragment mappings, with the exDNA sequences significantly reflecting the different active metabolism of yeast under the two conditions, either fermentative or respiratory.

The DNA fragments recovered from EFC were shorter and not overlapping with the significantly larger contigs found in LFC. Instead, the DNA fragments recovered for early EFC (at 6 h, when cells were still under respiratory metabolism) resulted to be included in the larger set of contigs reconstructed from LFC, where a respiratory metabolism was maintained during the whole run, as consistently observed in different chromosomes.

Considering the total exDNA recovered from the supernatants, it is relevant that it did not represent the whole yeast genome, but only 5.6 and 39.2% for EFC and LFC, respectively. This evidence was indicative of an active release of the nucleic acid into the medium, excluding a derivation from cellular lysis.

Pioneer studies reported the accumulation of DNA circles inside yeast cells in association with cell aging process [32]. Later on, eccDNA was reported in yeast [33], ranging in size from 1 to 38 kb and corresponding to 22% of the *S. cerevisiae* genome. These results were extended to different yeast strains, also showing reduction of eccDNA after 15 cell divisions [35]. In this context, the circular DNA accumulation has been discussed as a general phenomenon in eukaryotic cells, with sequences arising from different parts of eukaryotic genomes [50, 51], and possibly involved in cell aging [52].

The comparison between the eccDNA [33] (recovered from respiratory cells after 48 h batch cultures) with the exDNA recovered from our cultivations clearly showed a significant similarity of sequences with the respiratory LFC, and a much reduced correspondence with the mostly fermentative EFC. This was consistent both when considering the whole sequencing products and, more interestingly, when focusing the analysis on known high copy eccDNA (rDNA, Telomeric Y prime, *CUP1, ENA, HXT6-7*) that are always recovered in the LFC, but only partially in EFC exDNA. Moreover, the comparison of eccDNA with the sequences from early respiratory EFC consistently showed an intermediate similarity, lower than that found with LFC, but higher than that observed with EFC.

Noteworthy, the bioinformatic analysis by KEGGS pathways showed that the fragments from LFC included sequences of genes of specific metabolic pathways associated with respiratory metabolism, that were mostly absent among the fragments from EFC.

Different mechanisms of production of free circulating DNA fragments have been reported, reviewing their different origins, such as necrosis, apoptosis, and active secretion by living cells. The relative frequency of the sequences in such fragments was correlated to the density of DNA packaging into chromatin, so being associated with genome transcription activity and to the levels of methylation [12]. These observations of over-represented active regions among extracellular fragments are very consistent with our results in yeast. In fact, the exDNA recovered from yeast media was significantly associated with regions containing genes involved with the specific active metabolism displayed during cultivation, thus suggesting that their production is from accessible chromatin in transcriptionally active regions. This is in agreement with the reported subliminal replication activity in these regions [53, 54].

The inhibitory effect of exDNA on yeast growth was confirmed by the addition of exhausted media from EFC and LFC to *de novo* batch cultures. This produced very significant growth inhibition, which, in parallel, was reduced by iterative extractions of DNA from the media by HAP adsorption. Moreover, higher inhibition levels were shown by purified DNA. Instead, no inhibition was found when cells were exposed to the genomic *S. cerevisiae* DNA. This finding indirectly shows that growth inhibition was not due to genomic DNA deriving from possible cell lysis during cultivations, thus remarking the role of ssDNA released by living cells. Noteworthy, both exhausted media and extracted DNA from the fermentative EFC, compared to the respiratory LFC, induced a higher inhibitory effect on cells of a batch fermentative culture, suggesting a metabolism specific effect.

The analysis of the cell cycle dynamics in our fed-batch cultures by FCM showed that the growth rate decline of yeast populations, in both EFC and LFC, affected the cell cycle dynamics in different ways. Indeed, in a batch culture, the well-known progression into the different phases of the cell cycle typically showed the entrance of the whole population in a G0 phase in correspondence of the growth arrest due to nutrient depletion [55]. Instead, our results highlight, on one hand, that in EFC the growth arrest was associated to both G0 and S phases, with the percentage of cells in the S phase stabilized at 40% of the total population. On the other hand, in LFC, the distribution in the S phase at the end of the run, was like a typical starved batch culture, presumably due to the co-occurrence of both nutrient depletion and inhibitor accumulation, as also suggested by the ^1^H NMR correlation analysis.

The anomalous level of S cells observed at the end of the EFC happened despite the maintenance of the continuous feeding, thus apparently reflecting the accumulation of the inhibitor in the medium, even in the absence of nutrients limitation. The peculiarity of this phenomenon was also highlighted by the observed bimodality of the cell population in S phase, which appeared in correspondence with the beginning of the growth rate decline, i.e., the flexus in the growth curve [31]. Indeed, this can be seen as an indicator of the onset of the exDNA sensing by the cells, that either remained blocked in an early stage of the S phase or progressed along the cell cycle ending up to a later stop in G0.

Experimental observations of S-phase arrest in a growing cell population have been reported [56–58]. The intra S-phase arrest in yeast growth are presumably due to stalling replication, triggering the intra-S phase checkpoint, and are generally due to replication stress that impairs the processivity of the normal replisome [59, 60]. It is well known that ssDNA may be the crucial effector in response to replication stress in yeast [61]. In addition, interestingly, it is known that a threshold level of ssDNA exists that determines DNA damage checkpoint activation, although the amount of ssDNA required to reach the threshold is not clear [62].

**Figure 6:**
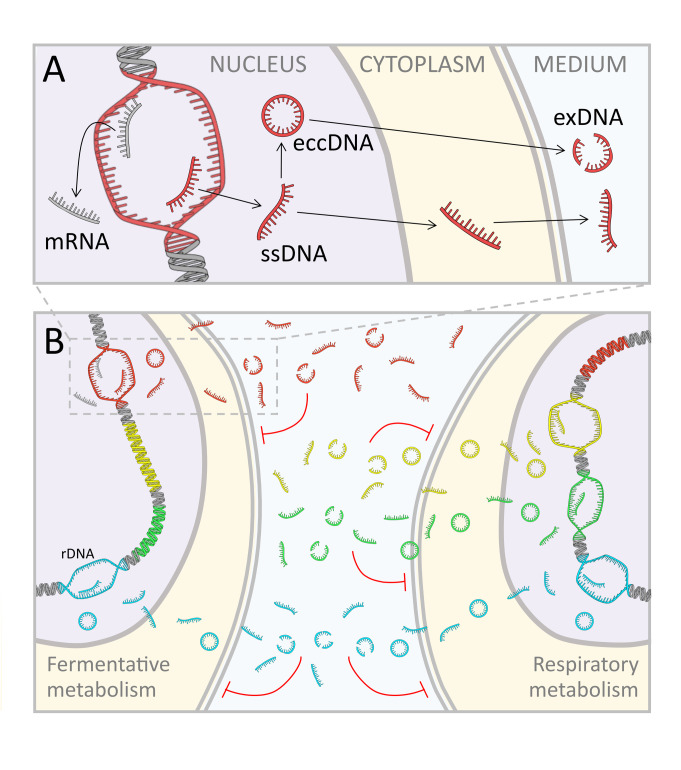
FIGURE 6: Model hypothesis of metabolism specific exDNA production and inhibitory effect in yeast cultures. **(A)** Production of single stranded DNA (ssDNA) fragments in the nucleus, with eccDNA formation, their moving through the cytoplasm, and release into the medium (exDNA). **(B)** The ssDNA production is associated to open transcription regions, so fragments that accumulate in the medium present specific sequences associated to the active metabolism. Respiratory cells produce a larger variety of exDNA compared to fermentative cells, whereas fragments from common regions of the genome, such as ribosomal DNA, are equally produced by all cells, irrespectively of their displayed metabolism. The inhibitory effect of exDNA is higher when cells are exposed to specific exDNA sequences corresponding to the active regions of the genome.

On the base of the above reported findings, we propose a new speculative model, which takes shape integrating in one conceptual picture the exDNA production and its inhibitory function, as schematically represented in **Fig. 6**: the production of free DNA fragments could be seen as the unavoidable side effect of unscheduled single stranded DNA synthesis within active transcription regions. This idea mirrors the recent finding that RNA transcription persists during DNA replication [54]. In other words, we suggest that, during active transcription, it is likely that nucleotide strands can be erroneously duplicated, thus producing fragments of ssDNA, which are fated to accumulate inside the cells and then continuously released into the extracellular environment. In this view, the eccDNA can be seen as the intermediate phase of the ongoing flow from the nucleus to the external environment, in which exDNA can be found at the end of the process.

As underlined, we found a large dominance of rDNA fragments in all samples of exDNA, independently of the metabolism displayed. This is consistent with the proposed mechanism because the rDNA is known to be present in multiple copies and the ribosome production is always active, under both fermentative and respiratory conditions. Work by Kobayashi and co-authors [63] described a phenomenon of instability of repeated ribosomal sequences, explaining the production of intracellular rDNA fragments, and interpreted this process as the driving mechanism of cellular aging.

The observed high heterogeneity of the sequences of exDNA supports the hypothesis that these fragments come from failing replicative initiations, thus providing waste-DNA replicons. This idea is further supported by the evident random distribution of the mismatches in the reads of exDNA that cannot be associated to the typical polymorphism of different haplotypes.

Therefore, the eccDNA found inside the cells and the exDNA recovered from the media appear to be different frames of a unique process, i.e., the active secretion of waste-DNA material. This is also in accordance with observations that secretion of exosomes in mammalian cells containing various lengths of chromosomal DNA fragments, maintains cellular homeostasis by removing harmful DNA from cells [64].

According to our model, although speculative and still requiring further experimental confirmation, the secretion of exDNA is a spontaneous process, leading to an emergent property at population level, that is a metabolism specific inhibition on cell proliferation. We suggest that ssDNA fragments when sensed as DNA damage, induce a quorum sensing action activating a regulatory cascade, leading to cell cycle arrest (**Fig. 6**).

In conclusion, the occurrence of exDNA in yeast cultures, its metabolism specificity, and the inhibitory effect on cell proliferation, paves the way to a new scenario of studies on actively secreted DNA by living cells. Further investigation is ongoing to elucidate the mechanism of action of the observed inhibitory effect of exDNA by both transcriptomic and metabolomic in depth analysis, coupled with an accurate mass and carbon balance. Also, the mechanism of release of exDNA in the culture medium is a topic of major relevance requiring future focused investigation in the context of vesicles trafficking at membrane level.

Moreover, in terms of application, it will be interesting to assess the relevance of exDNA as a self-produced inhibitory compound for its impact in the management of microbial cell cultures in bioreactors.

### Limitations of the study

One limitation of the present study is that experiments have been performed using only one strain of *S. cerevisiae,* the CEN.PK 2-1C, already studied in our previous work [31]. Here we used the same strain to identify the inhibitory compound. Indeed, this strain is considered as a reference strain belonging to the CEN.PK family of isogenic laboratory strains constructed with the expressed aim of meeting the requirements of physiologists, geneticists, and engineers [65]. However, further studies should extend the investigation to other *S. cerevisiae* strains and different yeast species.

A second limitation regarding the possible generality of our findings is that fed-batch cultures were done only with glucose as carbon and energy source to induce two different fundamental metabolisms. A larger variety of substrates and cultivation modes could allow to check the level of specificity of DNA secretion under other conditions.

## MATERIAL AND METHODS

### Experimental cultures

The strain selected for the study of growth inhibition during fed-batch cultures was CEN.PK2-1C (*MATa ura3-52 his3-D1 leu2-3,112 trp1-289 MAL2-8c SUC2*), purchased at EUROSCARF collection (www.uni-frankfurt.de/fb15/mikro/euroscarf).

Aerobic fed-batch cultures were performed in a 2.0 L working volume of a stirred fermenter, Bioflo 110 (New Brunswick Scientific) at 28°C. The vessel initially contained 1 L of a medium prepared [66] with 2% w/v glucose, vitamins and trace elements, and supplemented with 10 g L^−1^ of casamino acids (BD Bacto TM Casamino Acids, BectonDickinson & Co., Sparks, MD 21152 USA). Uracil, histidine, leucin, tryptophan were added to fully cover the yeast strain request for the entire fermentation run [67]. The reactor was inoculated with an adequate aliquot of a pre-culture in the exponential phase, to give an initial optical density at 590 nm (O.D._590_) of 0.04. After an initial batch phase (15 h), where glucose was depleted, the reactor was fed with a solution containing 50% w/v glucose and salts, trace elements, glutamic acid and vitamins [68].

Oxygen was supplied to the reactor by air sparging. The dissolved oxygen tension (DOT) was kept at 30% air saturation by the cascade system, by controlling the impeller speed, or, when this reached its maximum value (1,000 rpm) by enrichment with pure oxygen. The culture pH was maintained at 5.00 by automatic addition of 2N KOH during batch phase, and 10% v/v NH_4_OH during fed-batch phase. The foam level in the fermenter was controlled by the automatic addition of antifoam B (Sigma Aldrich) solution at 10% v/v.

After the initial batch phase (15 h), a fed-batch culture was performed by supplying the reactor with two types of feeding regimes, both starting with an exponentially increasing feeding at a specific feed rate (SFR) of 0,16 h^−1^. Hereafter, we named the two types of fed-batch cultures “exponential feeding culture” (EFC) and “limited feeding culture” (LFC), respectively.

The exponential feeding was calculated according to:

*F*(*t*)= *F*_*0*_ ·exp (*SFR*·*t*)

where *F*(*t*) is the time-dependent feed rate, *F*_*0*_ is the initial feed rate and SFR is the specific feed rate (h^−1^) which, in ideal conditions [69], is equal to the population specific growth rate (*µ*). The *SFR* value was below the *µ* critical value for the selected strain in correspondence of which ethanol begins to be produced [65].

In EFC, the exponential feeding was maintained all over the run. Differently, in LFC, a similar starting feeding regime (Eq. 1) was applied, followed by a logistically decreasing *SFR* value. In detail, the *SFR* starting from the initial value of 0,16 h^-1^ was progressively decreased, so to follow the specific growth rate (*µ)* decline of the culture, as already reported [31].

The panel A in Figure S1 shows the two different feeding profiles in EFC and LFC.

Samples of culture medium were collected from the reactor at different times (from 0 to 15 h of the batch phase, and from 0 to 30 h of the feeding phase) during the two experimental runs. Supernatants were recovered after centrifugation (3000 g, 5 min) followed by filtration (Stericup® Millipore Express®, 0,22 µm diameter), and used for analytical determinations of ethanol. Supernatants from the feeding phase were used for extraction of DNA and for ^1^H NMR analysis as described below. Supernatants at the end of EFC and LFC runs (exhausted media) were also used for experiments of inhibition of yeast growth.

Yeast cell mass was determined by O.D._590_ and dry weight determination. The calibration curve relating O.D._590_ values to biomass density provided a correlation factor of 2.30 O.D._590_ per g L^−1^. Absence of loss in cell viability during fed-batch runs was determined by viable count (in triplicate) on YPD (yeast extract 1%, bactopeptone 2%, dextrose 2% w/v) agar plates, incubated at 28°C for 48 h.

Ethanol in the supernatants were determined by Ethanol-enzymatic kit from Megazyme (Megazyme International, Ireland Ltd.). No ethanol was present in the exhausted medium from LFC, as expected. Instead, ethanol in the EFC exhausted medium was reduced down to 0,03% v/v, following vacuum distillation at 37°C using a Buchi R-215 (Büchi Labortechnik AG, Switzerland) rotary evaporator.

### ^1^H NMR characterization of the culture supernatants

^1^H NMR spectra were acquired for samples of supernatants collected from both EFC and LFC during the feeding phase at 8, 10, 12, 14, 16, 18, 20, 22 and 30 h and of the nutrient medium before yeast inoculation.

Samples (1 mL each) were centrifuged (3000 g, 5 min), lyophilized and kept at -20°C for ^1^H NMR analysis. Experiments were performed in triplicate to assure their reproducibility. ^1^H NMR spectra were acquired after solubilization of the dry residue with 600 µL of deuterium oxide (99,8% D_2_O) and transferred into a 5 mm NMR tube. DSS (Sodium trimethylsilylpropanesulfonate) was used as internal standard and the pH was adjusted to 6.0 by using KH_2_PO_4_ as a buffering agent and 1N NaOD [70]. The spectra were acquired at 298K on a Bruker Avance NEO 600 MHz spectrometer. The recycle time was set to 5 s, and a 45-pulse angle was used. Chemical shifts were referred to DSS signal (0.00 ppm) and the residual solvent signal for D_2_O resonated at 4.70 ppm. All spectra were processed using ^1^H NMR program (www.inmr.net), phased and baseline corrected manually. Spectral peak assignments of nucleotides were obtained by comparison with data reported in the literature [71] and standard nucleotides, purchased by Sigma-Aldrich, registered in our laboratory.

To obtain quantitative information of the detected compounds, the ^1^H NMR spectra were preliminarily normalized and reduced to integrated regions of equal widths (bins = 0.01 ppm), corresponding to 0-10 ppm and subsequently converted to ASCII files using the software package ^1^H NMR v. 5.1.2 (Mestrelab Research, Molfetta, Italy). Peak quantification was performed by signal integration relative to the internal standard, with peak intensity expressed as parts per thousand with respect to the whole spectrum once the region of the residual solvent peaks was excluded.

### Extraction of exDNA from culture supernatants

exDNA was directly extracted and purified from aliquots (5 mL) of filtered supernatants collected at different time during the feeding phase of both EFC and LFC. DNA extraction kit particularly indicated for complex matrices (DNeasy® PowerMax® Soil Kit, Qiagen, USA) was used following the manufacturer instructions. Briefly, each aliquot was placed in a 50 mL falcon tube containing beads (0.7 mm diameter) and a lysis buffer on a rotating mixer (40 rpm) for 30 min. After centrifugation (4°C, 3 min, 2500 g) the supernatant was collected and subjected to 5 cycles of purification and centrifugation to precipitate additional non-DNA organic and inorganic compounds (including cell debris and proteins). At the end of purification, all DNA samples were suspended in 5 mL H_2_O and quantified. Purity of the samples was checked verifying the ratios A_260_:A_280_ and A_260_:A_230_ by Nanodrop spectrophotometer (Thermo Fisher Scientific, Waltham, Massachusetts, USA).

Furthermore, aiming to confirm the occurrence of exDNA in the culture medium, a different procedure of extraction was followed in the case of the exhausted medium of EFC, i.e. the conventional operating mode for fed-batch systems. This was made by adsorption of the exhausted medium on hydroxyapatite (HAP) column, according to Anker *et al.* (1975) [15] with some modifications described below. The medium was loaded on a HAP (Hydroxyapatite DNA grade: Bio-Gel HTP) column, which was previously adapted in 0.005 M phosphate buffer pH 6.8, pre-heated at 60°C. The column was eluted with 0.005 M PBS and the collected fraction was called A. Single-stranded DNA (ssDNA) was eluted from the column with 0.12 M phosphate buffer and called B, while the double-stranded DNA (dsDNA) was eluted from the column with 0.48 M phosphate buffer and called C. The column was then equilibrated with 0.005 M phosphate buffer. The extraction procedure by HAP of exDNA from the exhausted medium was repeated nine consecutive times collecting all intermediate A fractions (excluded volumes after passage through the column), which were used for *de novo* inhibitory experiments on yeast growth. Fractions B and C were used to determine the amount of ssDNA (ng μL^-1^) and dsDNA (ng μL^-1^).

DNA was quantified by fluorimeter Qubit™ 3.0 using Qubit dsDNA HS Assay Kit, and Qubit ssDNA Assay Kit, (Life Technology, Carlsbad, California, USA). Also, RNA was detected in the samples by Qubit RNA BR Assay Kit (Life Technology, Carlsbad, California, USA).

The quality of samples was assessed by NanoDrop spectrophotometer (Thermo Fisher Scientific, Waltham, Massachusetts, USA). Fluorometric quantification assays were made on all samples to determine the amount of ssDNA (ng μL^-1^), dsDNA (ng μL^-1^), and RNA (ng μL^-1^).

### Sequencing of exDNA and bioinformatic analysis of exDNA

In the case of EFC, samples of supernatants were collected at 6 and 30 h when the culture exhibited either respiratory or fermentative metabolism, respectively. Instead LFC supernatant was sampled only at 30 h, since the respiratory metabolism was maintained constant across the whole run. The collected supernatants from two biological replicates were directly subjected to amplification by using REPLI-g kit (Quiagen) that operates a random amplification with minimal sequence bias on both ssDNA and dsDNA.

The amplification products (EFC 6_h, EFC and LFC) were sequenced by the IlluminaNextSeq500 1X150 (single-read). The raw reads per sample were cleaned from adaptors and low-quality bases using the Trim Galore package (http://www.bioinformatics.babraham.ac.uk/projects/trim_galore/), applying the default settings for single read sequencing. The cleaned reads were then mapped onto the Yeast genome (S288C, r64) downloaded from the Saccharomyces Genome Database using the STAR software (version 2.4.2a) [72] and accepting up to 50 mismatches.

The map of the reads versus the genome was performed following two different criteria by reporting either “multiple” hits (to detect all matches that each read can find on the genome) or reads with “unique” hits on the genome. The sequence analysis to assess the specificity of exDNA sequences recovered from the supernatants was performed by the BLAST tool versus the nucleotide sequence database (GenBank) at the NCBI web site.

### Comparative analysis of exDNA vs. eccDNA

To assess the degree of similarity among DNA fragments accumulating in the exhausted media, in EFC 6 h supernatant, and those occurring in the yeast cells collected after 72 h of a batch culture, i.e., under respiratory metabolism [33], two datasets were used. They included: (i) the results of high throughput DNA sequencing, as well as alignment on yeast genome, of EFC and LFC fragments (from exhausted media and EFC 6 h), (three different replicated samples; Supplemental Dataset S1); and (ii) the extrachromosomal circular DNA (eccDNA) [33].

In both datasets, the location on the genome (reference SGD), the length in bp and the abundance (number of reads, standardized and expressed as FPKM), as well as the list of genes completely or partially overlapping to each DNA fragment found in the samples are reported.

Therefore, the comparative analysis was carried out considering three different metrics of similarity among the samples: a) the number of bp in common between the two samples (i.e. aligning at the same position on the reference yeast genome); b) the number of overlapping contigs between the two samples; c) the number of genes in common between the two samples. All metrics were expressed as raw values or as percentages of the total numbers in either sample considered in the comparison. Results were presented as tables or, in the case of gene numbers, as Venn's diagrams.

Moreover, the list of genes of the pathways associated to yeast respiration or common to both respiration and fermentation (genes of cell cycle and glycolysis/gluconeogenesis) was obtained from KEGG database (https://www.kegg.jp/kegg/) in the KEGG pathways section (https://www.kegg.jp/kegg/pathway.html).

### Inhibitory effect of exhausted media and exDNA on yeast growth

The inhibitory effects of both exhausted media (also after passage through the HAP column) and extracted exDNA were tested on yeast growth in batch cultures. Aiming at this, batch cultures were set up in 50 or 5 cm^-3^ shake flasks (10 or 1 mL final working volumes), in the case of tests with exhausted media or exDNA, respectively. Flasks contained the same mineral medium used for fed-batch cultivations, with 2% glucose, supplemented with casamino acids, uracil, histidine, leucin, tryptophan, vitamins and trace elements, as reported above. In the case of the inhibitory tests with exhausted media, the latter were added to the substrate up to a final concentration of 75% v/v. In the experiments with exDNA from EFC and LFC, the purified nucleic acid was added so to have a final concentration of 3.0 ng µL^-1^, which was the concentration found at the end of the fed-batch runs. As heterologous DNA (nonself-DNA), a commercial fish sperm ssDNA (Roche Diagnostics, Netherlands) provided as a pure, sonicated, denatured single-stranded DNA fragment mixture (chain length 120 to 3,000 nucleotides) was used at the same final concentration of 3.0 ng µL^-1^. In addition, another heterologous DNA, genomic DNA extracted from *Candida albicans* ATCC 90028 was used. Moreover, a genomic DNA of *S. cerevisiae* CEN.PK 2-1C was also used in the inhibition test. Both genomic DNAs were prepared from 24 h cells grown in YPD medium at 30°C, following the standardized protocol already reported [26]. Both genomic DNAs were used at 30 ng µL^-1^ final concentrations in the inhibition test.

Pre-treatment of exDNA with S1 nuclease (Promega Co., Madison WI, USA) or with DNAse (Promega) was performed incubating the exDNA samples in the presence of the enzyme at 37°C for 2 h, following the manufacturer's instructions.

Supplementary experiments were also performed adding the amplification product obtained by Repli-g from EFC in inhibitory experiments on yeast growth. The amplification product was sonicated [26] and added to *de novo* batch cultures at concentration ranging from 10 to 80 ng µL^-1.^ Pre-treatment of such amplified DNA with DNase (Promega) was performed incubating DNA in the presence of the enzyme following manufacturer's instructions.

Also, RNA extracted from *S. cerevisiae* CEN.PK2-1C strain, in parallel with a heterologous RNA from human Hematopoietic Progenitor Cells (HPC) available in the laboratory were used in inhibition tests. Both RNAs were extracted by using RNeasy micro kit (Qiagen) following the manufacturer's instructions., and used at final concentrations of 3, 15 and 30 ng µL^-1^.

Flasks were inoculated with an adequate aliquot of a yeast pre-culture, to give an initial O.D._590_ of 0.1 and incubated at 28°C, 200 rpm. Yeast growth was checked after 12 h incubation by determining cell density (O.D._590_). Data are mean values of O.D._590_ assessed after the incubation time expressed as percentage of untreated control (=100).

### Flow Cytometric Analysis (FCM)

The cell cycle dynamics was studied during the feeding phases of the two fed-batch runs, EFC and LFC, by assessing the DNA content in the yeast cells by cytofluorimetric analysis. Aiming at this, yeast cell samples, collected from the bioreactor at different times during the feeding phase of both EFC and LFC, were centrifuged (3000 g, 5 min) to obtain cell pellets. Then, cells were re-suspended and fixed in 75% v/v ethanol, added dropwise under continuous vortexing to avoid cell agglomeration.

Fixed cells were centrifuged, treated with 1 mg mL^-1^ DNase-free RNAse A (Sigma-Aldrich) and stained with SYTOX Green (1 µM, Invitrogen™, λex 504 nm/λem 523 nm) as DNA binding dye [73]. Cells were acquired by Gallios Flow cytometer, equipped with three lasers (405 nm, 488 nm, 633 nm, Beckman Coulter, Milan, Italy) and data were analysed with Kaluza Analysis Software v. 2.1 (Beckman Coulter).

In parallel, from a batch culture set up at 28°C with the same culture medium [66, 67], yeast cells were collected at 0.D._590_ = 0,6 (exponential cells) and after seven days (starved cells), to be used as reference samples. Cells collected from the batch culture were processed for flow cytometric analysis as reported above.

### Statistical analysis

An extensive correlation analysis [74] was performed considering the linear correlation (Pearson's *r*) between yeast growth rate (i.e. Δ mass/Δ t, with Δ t = *t*_i_-*t*_i-1_ at each *i*-th observation time) in the growing media and ^1^H NMR data recorded for the same media at each resonance signal (n = 1000, from 0.01 ppm t 10 ppm with interval 0.01 ppm), providing a fine-resolution profile of the variation in ^1^H types in the tested media with its associated effect on yeast growth. In this way, a significant correlation, either positive or negative, indicated a causal effect, either enhancing or inhibiting on yeast growth, respectively, specifically attributable to the molecular trait of the medium corresponding to the ^1^H NMR signal. Two threshold levels were considered for significant correlations (p< 0.001 and p< 0.05).

Considering the bioassays testing the effects of exhausted media, DNA purified form the same media, and heterologous DNA on yeast growth, a one-way ANOVA was run on the dependent variable expressed in percentage of the untreated control, considering the treatment as the dependent variable (five levels: EFC and LFC media, EFC ad LFC DNA, heterologous DNA). Pairwise significant differences among treatments were assessed by Tuckey posthoc test at α = 0.05.

### Materials and resources

Details on all used reagents, yeast strains and software can be found in Table S2.

## AUTHOR CONTRIBUTION

EdA design and coordination of all experimental work, conceptual discussion, manuscript writing. GI conceptual discussion, data analysis (comparison of sequences of exDNA and eccDNA, correlation of NMR spectral data). FC conceptual discussion, manuscript and figures preparation. MLC coordination of sequencing and bioinformatic analysis. CC data analysis and figures of exDNA sequences. EP experimental work of inhibition tests, KEGG pathway analysis of exDNA sequences. PT and AF DNA extraction and inhibition tests. GB and FM critical discussion and manuscript revision. RC critical discussion and cell cycle analysis. MI DNA amplification and inhibition tests. CL and PP fed-batch cultures and bioreactors setting. MS and VT flow cytometric analysis and cell cycle data analysis. BdF HAP exDNA extraction and quantification. VL coordination of NMR analysis. SM team coordination, conceptual design of the work and manuscript preparation.

## SUPPLEMENTAL MATERIAL

Click here for supplemental data file.

Click here for supplemental data file.

Click here for supplemental data file.

All supplemental data for this article are available online at https://www.microbialcell.com/researcharticles/2023a-de-alteriis-microbial-cell/.

## Abbreviations:

eccDNA: – extrachromosomal circular DNA,
EFC: – exponential feeding culture,
exDNA: – extracellular DNA,
FCM: – flow cytometric analysis,
LFC: – limited feeding culture.

## References

[1] Ascher J, Ceccherini MT, Pantani OL, Agnelli A, Borgogni F, Guerri G, Nannipieri P, Pietramellara G (2009). Sequential extraction and genetic fingerprinting of a forest soil metagenome.. Appl Soil Ecol.

[2] Nagler M, Podmirseg SM, Griffith GW, Insam H, Ascher-Jenull J (2018). The use of extracellular DNA as a proxy for specific microbial activity.. Appl Microbiol Biotechnol.

[3] Torti A, Lever MA, Jørgensen BB (2015). Origin, dynamics, and implications of extracellular DNA pools in marine sediments.. Mar Genomics.

[4] Monticolo F, Palomba E, Termolino P, Chiaiese P, de Alteriis E, Mazzoleni S, Chiusano ML (2020). The Role of DNA in the Extracellular Environment: A Focus on NETs, RETs and Biofilms.. Front Plant Sci.

[5] Yang K, Wang L, Cao X, Gu Z, Zhao G, Ran M, Yan Y, Yan J, Xu L, Gao C, Yang M (2022). The Origin, Function, Distribution, Quantification, and Research Advances of Extracellular DNA.. Int J Mol Sci.

[6] Zarnowski R, Westler WM, Lacmbouh GA, Marita JM, Bothe JR, Bernhardt J, Lounes-Hadj Sahraoui A, Fontaine J, Sanchez H, Hatfield RD, Ntambi JM, Nett JE, Mitchell AP, Andes DR (2014). Novel entries in a fungal biofilm matrix encyclopedia.. MBio.

[7] Flemming H-C, van Hullebusch ED, Neu TR, Nielsen PH, Seviour T, Stoodley P, Wingender J, Wuertz S (2022). The biofilm matrix: multitasking in a shared space.. Nat Rev Microbiol.

[8] Tamkovich S, Laktionov P (2019). Cell-surface-bound circulating DNA in the blood: Biology and clinical application.. IUBMB Life.

[9] Brinkmann V, Reichard U, Goosmann C, Fauler B, Uhlemann Y, Weiss DS, Weinrauch Y, Zychlinsky A (2004). Neutrophil extracellular traps kill bacteria.. Science.

[10] Hawes MC, Curlango-Rivera G, Wen F, White GJ, Vanetten HD, Xiong Z (2011). Extracellular DNA: the tip of root defenses?. Plant Sci.

[11] Wen F, Curlango-Rivera G, Huskey DA, Xiong Z, Hawes MC (2017). Visualization of extracellular DNA released during border cell separation from the root cap.. Am J Bot.

[12] Rykova EY, Morozkin ES, Ponomaryova AA, Loseva EM, Zaporozhchenko IA, Cherdyntseva N V, Vlassov V V, Laktionov PP (2012). Cell-free and cell-bound circulating nucleic acid complexes: mechanisms of generation, concentration and content.. Expert Opin Biol Ther.

[13] Thierry AR, El Messaoudi S, Gahan PB, Anker P, Stroun M (2016). Origins, structures, and functions of circulating DNA in oncology.. Cancer Metastasis Rev.

[14] Aucamp J, Bronkhorst AJ, Badenhorst CPS, Pretorius PJ (2018). The diverse origins of circulating cell-free DNA in the human body: a critical re-evaluation of the literature.. Biol Rev Camb Philos Soc.

[15] Anker P, Stroun M, Maurice PA (1975). Spontaneous release of DNA by human blood lymphocytes as shown in an in vitro system.. Cancer Res.

[16] Kustanovich A, Schwartz R, Peretz T, Grinshpun A (2019). Life and death of circulating cell-free DNA.. Cancer Biol Ther.

[17] Kalluri R, LeBleu VS (2016). Discovery of Double-Stranded Genomic DNA in Circulating Exosomes.. Cold Spring Harb Symp Quant Biol.

[18] de Aldecoa AL, Zafra O, González-Pastor JE (2017). Mechanisms and Regulation of Extracellular DNA Release and Its Biological Roles in Microbial Communities.. Front Microbiol.

[19] Draghi JA, Turner PE (2006). DNA secretion and gene-level selection in bacteria.. Microbiology.

[20] Barnes AMT, Ballering KS, Leibman RS, Wells CL, Dunny GM (2012). Enterococcus faecalis produces abundant extracellular structures containing DNA in the absence of cell lysis during early biofilm formation.. MBio.

[21] Schwartz K, Ganesan M, Payne DE, Solomon MJ, Boles BR (2016). Extracellular DNA facilitates the formation of functional amyloids in Staphylococcus aureus biofilms.. Mol Microbiol.

[22] Mazzoleni S, Bonanomi G, Incerti G, Chiusano ML, Termolino P, Mingo A, Senatore M, Giannino F, Cartenì F, Rietkerk M, Lanzotti V (2015). Inhibitory and toxic effects of extracellular self-DNA in litter: a mechanism for negative plant–soil feedbacks?. New Phytol.

[23] Mazzoleni S, Cartenì F, Bonanomi G, Senatore M, Termolino P, Giannino F, Incerti G, Rietkerk M, Lanzotti V, Chiusano ML (2015). Inhibitory effects of extracellular self-DNA: a general biological process?. New Phytol.

[24] Palomba E, Chiaiese P, Termolino P, Paparo R, Filippone E, Mazzoleni S, Chiusano ML (2022). Effects of Extracellular Self- and Nonself-DNA on the Freshwater Microalga Chlamydomonas reinhardtii and on the Marine Microalga Nannochloropsis gaditana.. Plants.

[25] Germoglio M, Adamo A, Incerti G, Cartenì F, Gigliotti S, Storlazzi A, Mazzoleni S (2022). Self-DNA Exposure Induces Developmental Defects and Germline DNA Damage Response in Caenorhabditis elegans.. Biology.

[26] Chiusano ML, Incerti G, Colantuono C, Termolino P, Palomba E, Monticolo F, Benvenuto G, Foscari A, Esposito A, Marti L, de Lorenzo G, Vega-Muñoz I, Heil M, Carteni F, Bonanomi G, Mazzoleni S (2021). Arabidopsis thaliana Response to Extracellular DNA: Self Versus Nonself Exposure.. Plants.

[27] Lanzotti V, Grauso L, Mangoni A, Termolino P, Palomba E, Anzano A, Incerti G, Mazzoleni S (2022). Metabolomics and molecular networking analyses in Arabidopsis thaliana show that extracellular self-DNA affects nucleoside/nucleotide cycles with accumulation of cAMP, cGMP and N6-methyl-AMP.. Phytochemistry.

[28] Pérez-Torrado R, Matallana E (2015). Enhanced fermentative capacity of yeasts engineered in storage carbohydrate metabolism.. Biotechnol Prog.

[29] Mendoza-Vega O, Sabatié J, Brown SW (1994). Industrial production of heterologous proteins by fed-batch cultures of the yeast Saccharomyces cerevisiae.. FEMS Microbiol Rev.

[30] Mattanovich D, Branduardi P, Dato L, Gasser B, Sauer M, Porro D (2012). Recombinant protein production in yeasts.. Methods Mol Biol.

[31] Mazzoleni S, Landi C, Cartenì F, de Alteriis E, Giannino F, Paciello L, Parascandola P (2015). A novel process-based model of microbial growth: self-inhibition in Saccharomyces cerevisiae aerobic fed-batch cultures.. Microb Cell Fact.

[32] Sinclair DA, Guarente L (1997). Extrachromosomal rDNA circles–a cause of aging in yeast.. Cell.

[33] Møller HD, Parsons L, Jørgensen TS, Botstein D, Regenberg B (2015). Extrachromosomal circular DNA is common in yeast.. Proc Natl Acad Sci.

[34] Møller HD, Bojsen RK, Tachibana C, Parsons L, Botstein D, Regenberg B (2016). Genome-wide Purification of Extrachromosomal Circular DNA from Eukaryotic Cells.. J Vis Exp.

[35] Prada-Luengo I, Møller HD, Henriksen RA, Gao Q, Larsen CE, Alizadeh S, Maretty L, Houseley J, Regenberg B (2020). Replicative aging is associated with loss of genetic heterogeneity from extrachromosomal circular DNA in Saccharomyces cerevisiae.. Nucleic Acids Res.

[36] Fan TW-M (1996). Metabolite profiling by one- and two-dimensional NMR analysis of complex mixtures.. Prog Nucl Magn Reson Spectrosc.

[37] Ippel JH, Lanzotti V, Galeone A, Mayol L, Van den Boogaart JE, Pikkemaat JA, Altona C (1995). Thermodynamics of melting of the circular dumbbell d<pCGC-TT-GCG-TT>.. Biopolymers.

[38] Monod J (1949). THE GROWTH OF BACTERIAL CULTURES.. Annu Rev Microbiol.

[39] Riesenberg D, Guthke R (1999). High-cell-density cultivation of microorganisms.. Appl Microbiol Biotechnol.

[40] van Hoek P, de Hulster E, van Dijken JP, Pronk JT (2000). Fermentative capacity in high-cell-density fed-batch cultures of baker's yeast.. Biotechnol Bioeng.

[41] Shiloach J, Fass R (2005). Growing E. coli to high cell density–a historical perspective on method development.. Biotechnol Adv.

[42] Riesenberg D (1991). High-cell-density cultivation of Escherichia coli.. Curr Opin Biotechnol.

[43] Landi C, Paciello L, de Alteriis E, Brambilla L, Parascandola P (2015). High cell density culture with S. cerevisiae CEN.PK113-5D for IL-1β production: optimization, modeling, and physiological aspects.. Bioprocess Biosyst Eng.

[44] Lee SY (1996). High cell-density culture of Escherichia coli.. Trends Biotechnol.

[45] De Deken RH (1966). The Crabtree Effect: A Regulatory System in Yeast.. Microbiology.

[46] de Alteriis E, Cartenì F, Parascandola P, Serpa J, Mazzoleni S (2018). Revisiting the Crabtree/Warburg effect in a dynamic perspective: a fitness advantage against sugar-induced cell death.. Cell Cycle.

[47] Avbelj M, Zupan J, Kranjc L, Raspor P (2015). Quorum-Sensing Kinetics in Saccharomyces cerevisiae: A Symphony of ARO Genes and Aromatic Alcohols.. J Agric Food Chem.

[48] Barriuso J, Hogan DA, Keshavarz T, Martínez MJ (2018). Role of quorum sensing and chemical communication in fungal biotechnology and pathogenesis.. FEMS Microbiol Rev.

[49] Tang L, Schramm A, Neu TR, Revsbech NP, Meyer RL (2013). Extracellular DNA in adhesion and biofilm formation of four environmental isolates: a quantitative study.. FEMS Microbiol Ecol.

[50] Arrey G, Keating ST, Regenberg B (2022). A unifying model for extrachromosomal circular DNA load in eukaryotic cells.. Semin Cell Dev Biol.

[51] Møller HD, Mohiyuddin M, Prada-Luengo I, Sailani MR, Halling JF, Plomgaard P, Maretty L, Hansen AJ, Snyder MP, Pilegaard H, Lam HYK, Regenberg B (2018). Circular DNA elements of chromosomal origin are common in healthy human somatic tissue.. Nat Commun.

[52] Hull RM, Houseley J (2020). The adaptive potential of circular DNA accumulation in ageing cells.. Curr Genet.

[53] Saponaro M (2022). Transcription-Replication Coordination.. Life.

[54] Wang J, Rojas P, Mao J, Mustè Sadurnì M, Garnier O, Xiao S, Higgs MR, Garcia P, Saponaro M (2021). Persistence of RNA transcription during DNA replication delays duplication of transcription start sites until G2/M.. Cell Rep.

[55] Delobel P, Tesnière C (2014). A simple FCM method to avoid misinterpretation in Saccharomyces cerevisiae cell cycle assessment between G0 and sub-G1.. PLoS One.

[56] Ciardo D, Goldar A, Marheineke K (2019). On the Interplay of the DNA Replication Program and the Intra-S Phase Checkpoint Pathway.. Genes.

[57] Segurado M, Tercero JA (2009). The S-phase checkpoint: targeting the replication fork.. Biol Cell.

[58] Paulovich AG, Hartwell LH (1995). A checkpoint regulates the rate of progression through S phase in S. cerevisiae in response to DNA damage.. Cell.

[59] Pardo B, Crabbé L, Pasero P (2017). Signaling pathways of replication stress in yeast.. FEMS Yeast Res.

[60] Sabatinos SA, Forsburg SL (2015). Managing Single-Stranded DNA during Replication Stress in Fission Yeast.. Biomolecules.

[61] Bantele SCS, Lisby M, Pfander B (2019). Quantitative sensing and signalling of single-stranded DNA during the DNA damage response.. Nat Commun.

[62] Toledo L, Neelsen KJ, Lukas J (2017). Replication Catastrophe: When a Checkpoint Fails because of Exhaustion.. Mol Cell.

[63] Ganley ARD, Kobayashi T (2014). Ribosomal DNA and cellular senescence: new evidence supporting the connection between rDNA and aging.. FEMS Yeast Res.

[64] Takahashi A, Okada R, Nagao K, Kawamata Y, Hanyu A, Yoshimoto S, Takasugi M, Watanabe S, Kanemaki MT, Obuse C, Hara E (2017). Exosomes maintain cellular homeostasis by excreting harmful DNA from cells.. Nat Commun.

[65] van Dijken JP, Bauer J, Brambilla L, Duboc P, Francois JM, Gancedo C, Giuseppin ML, Heijnen JJ, Hoare M, Lange HC, Madden EA, Niederberger P, Nielsen J, Parrou JL, Petit T, Porro D, Reuss M, N van R, Rizzi M, Steensma HY, Verrips CT, Vindeløv J, Pronk JT (2000). An interlaboratory comparison of physiological and genetic properties of four Saccharomyces cerevisiae strains.. Enzyme Microb Technol.

[66] Verduyn C, Postma E, Scheffers WA, Van Dijken JP (1992). Effect of benzoic acid on metabolic fluxes in yeasts: a continuous-culture study on the regulation of respiration and alcoholic fermentation.. Yeast.

[67] Pronk JT (2002). Auxotrophic yeast strains in fundamental and applied research.. Appl Environ Microbiol.

[68] Paciello L, de Alteriis E, Mazzoni C, Palermo V, Zueco J, Parascandola P (2009). Performance of the auxotrophic Saccharomyces cerevisiae BY4741 as host for the production of IL-1β in aerated fed-batch reactor: role of ACA supplementation, strain viability, and maintenance energy.. Microb Cell Fact.

[69] Enfors S-O, Häggström L (1992).

[70] de Falco B, Incerti G, Bochicchio R, Phillips TD, Amato M, Lanzotti V (2017). Metabolomic analysis of Salvia hispanica seeds using NMR spectroscopy and multivariate data analysis.. Ind Crops Prod.

[71] Scheek RM, Boelens R, Russo N, Van Boom JH, Kaptein R (1984). Sequential resonance assignments in proton NMR spectra of oligonucleotides by two-dimensional NMR spectroscopy.. Biochemistry.

[72] Robinson MD, McCarthy DJ, Smyth GK (2010). edgeR: a Bioconductor package for differential expression analysis of digital gene expression data.. Bioinformatics.

[73] Palomba E, Tirelli V, de Alteriis E, Parascandola P, Landi C, Mazzoleni S, Sanchez M (2021). A cytofluorimetric analysis of a Saccharomyces cerevisiae population cultured in a fed-batch bioreactor.. PLoS One..

[74] Benjamini Y, Hochberg Y (1995). Controlling the False Discovery Rate: A Practical and Powerful Approach to Multiple Testing.. J R Stat Soc Ser B..

