# Supplementary material for: Extracellular DNA secreted in yeast cultures is metabolism-specific and inhibits cell proliferation
Source: Microb Cell. 2023 Nov 23;10(12):292–5. doi: 10.15698/mic2023.12.810 (PMC10695634; doi:10.15698/mic2023.12.810)
Supplement: Supplementary file 3 [file mic-10-277-s03.pdf]

Extracellular DNA secreted in yeast cultures is metabolism-specific and inhibits cell proliferation

Elisabetta de Alteriis, Guido Incerti, Fabrizio Cartenì, Maria Luisa Chiusano, Chiara Colantuono, Emanuela Palomba, Pasquale Termolino, Francesco Monticolo, Alfonso Esposito, Giuliano Bonanomi, Rosanna Capparelli, Marco Iannaccone, Alessandro Foscari, Carmine Landi, Palma Parascandola, Massimo Sanchez, Valentina Tirelli, Bruna de Falco, Virginia Lanzotti, Stefano Mazzoleni*

*Corresponding author: stefano.mazzoleni@unina.it

SUPPLEMENTARY MATERIALS

Figure S1. (A) Glucose feeding profiles in EFC and LFC fed-batch cultures. (B) Residual glucose concentrations in the medium of EFC and LFC.

Figure S2. ^{1}H NMR spectrum in D_2O of the yeast nutrient medium before inoculation.

Figure S3. Details of the ^{1}H NMR spectra in D_2O at different hours.

Figure S4. Electrophoretic mobility in agarose gel (1% w/v) of the DNA obtained by direct REPLIg amplification of EFC and LFC supernatants.

Figure S5. (A) Effect of different concentrations of DNA amplified from EFC supernatant on yeast growth in batch cultures. (B) Electrophoresis on 10% acrylamide gel of calf thymus DNA, DNA obtained by REPLIg amplification of EFC, and fish sperm DNA.

Figure S6. Examples of exDNA nucleotide reads mapped to the S288C *S. cerevisiae* reference genome using both “unique” and “multiple” approaches**.**

Figure S7. BLAST based alignment of three different reads mapping on the rDNA region (chromosome XII) *versus* the GenBank nucleotide database.

Figure S8. BLAST based alignment of three different reads mapping on the rDNA region (chromosome XII) *versus* the GenBank nucleotide database eliminating all possible hits matching versus “Saccharomyces” derived sequences, i.e., excluding all possible sequences referred to this term in the screened database.

Table S1. Results of two inhibition tests performed with *S. cerevisiae* CEN.PK1-2C treated with RNA extracted from either the same strain or human Hematopoietic Progenitor Cells (HPC).

Table S2. List of reagents, yeast strains and software used in this work.

Supplemental Dataset S1. Results of Illumina sequencing of exDNA purified from growth media of EFC, EFC 6h and LFC and the comparison with eccDNA from Møller *et al.* (2015). See attached Excel file ("Supplemental Dataset S1.xlsx").

Supplemental Dataset S2. Lists of genes partially or completely included in exDNA fragments from growing media (LFF, EFC, EFC6h) and, for comparison, in eccDNA from Moeller et al. (2015). See attached Excel file ("Supplemental Dataset S2.xlsx").

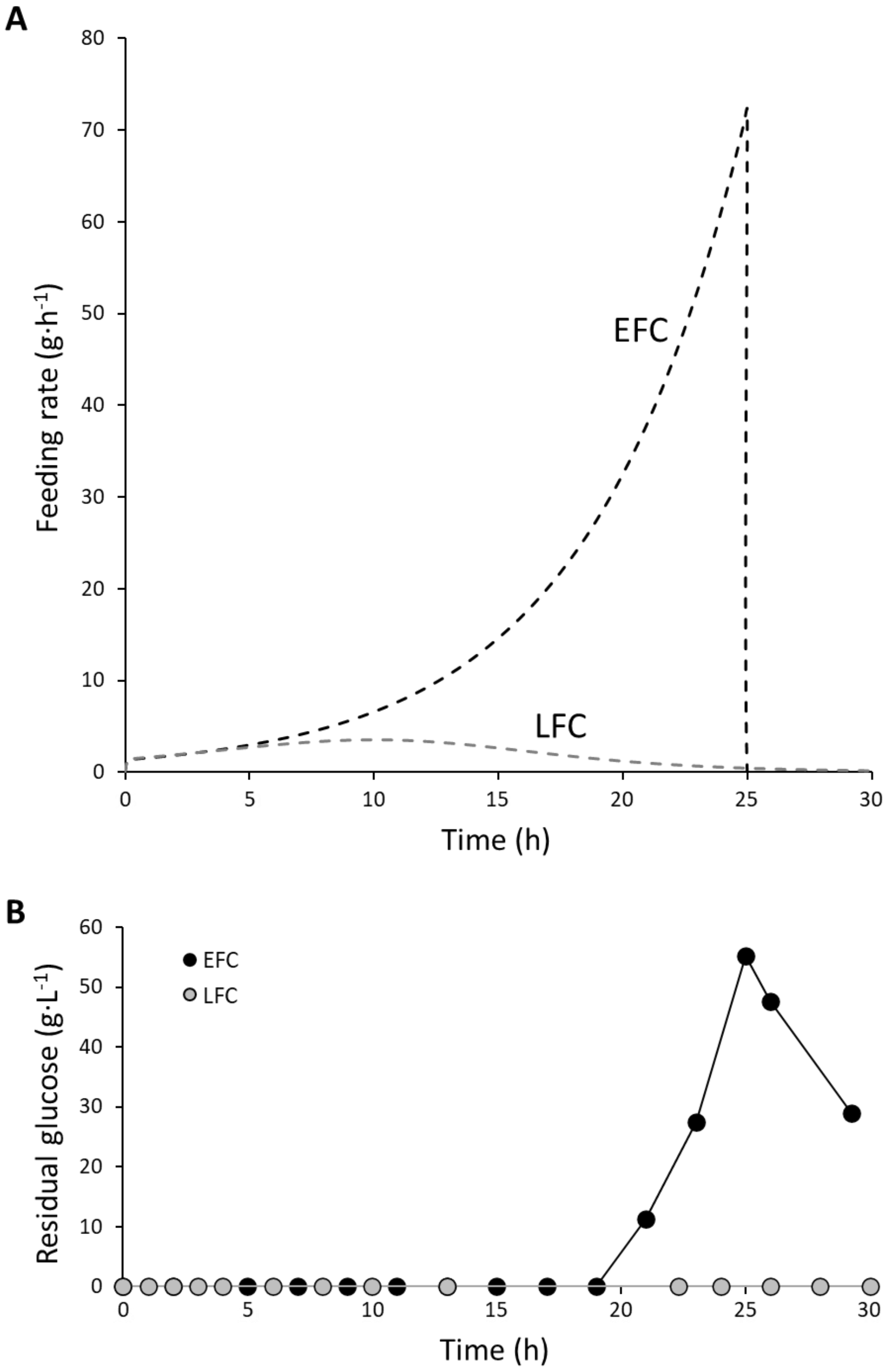

Figure S1. (A) Glucose feeding profiles in EFC and LFC fed-batch cultures. (B) Residual glucose concentrations in the medium of EFC and LFC.

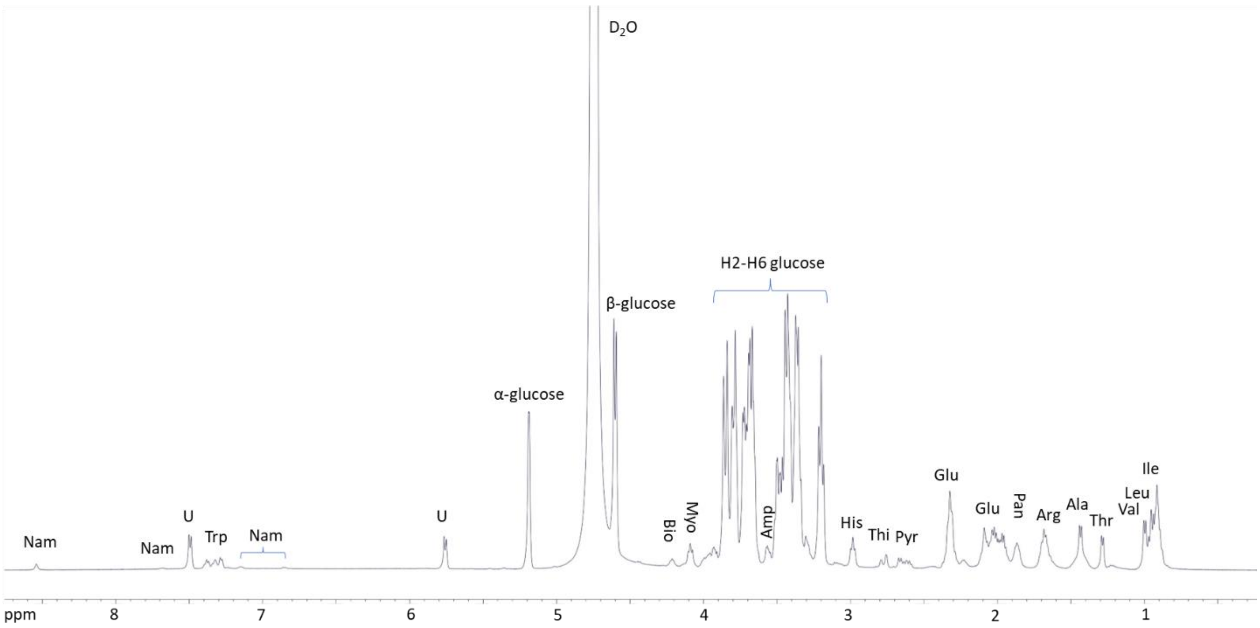

Figure S2. ^{1}H NMR spectrum in D_2O of the yeast nutrient medium before inoculation. Amp = ampicillin; Bio = biotin; Ala = Alanine; Arg = arginine; Glu = glutaminc acid; His = histidine; Ile= Isoleucine; Leu = leucine; Myo = myo-inositol; Nam = nicotinamide; Pan = calcium panthotenate; Pyr = pyridoxine hydrochloride; Thi= thiamine hydrochloride; Thr = threonine; Trp = tryptophan; U = uracyl; Val = valine.

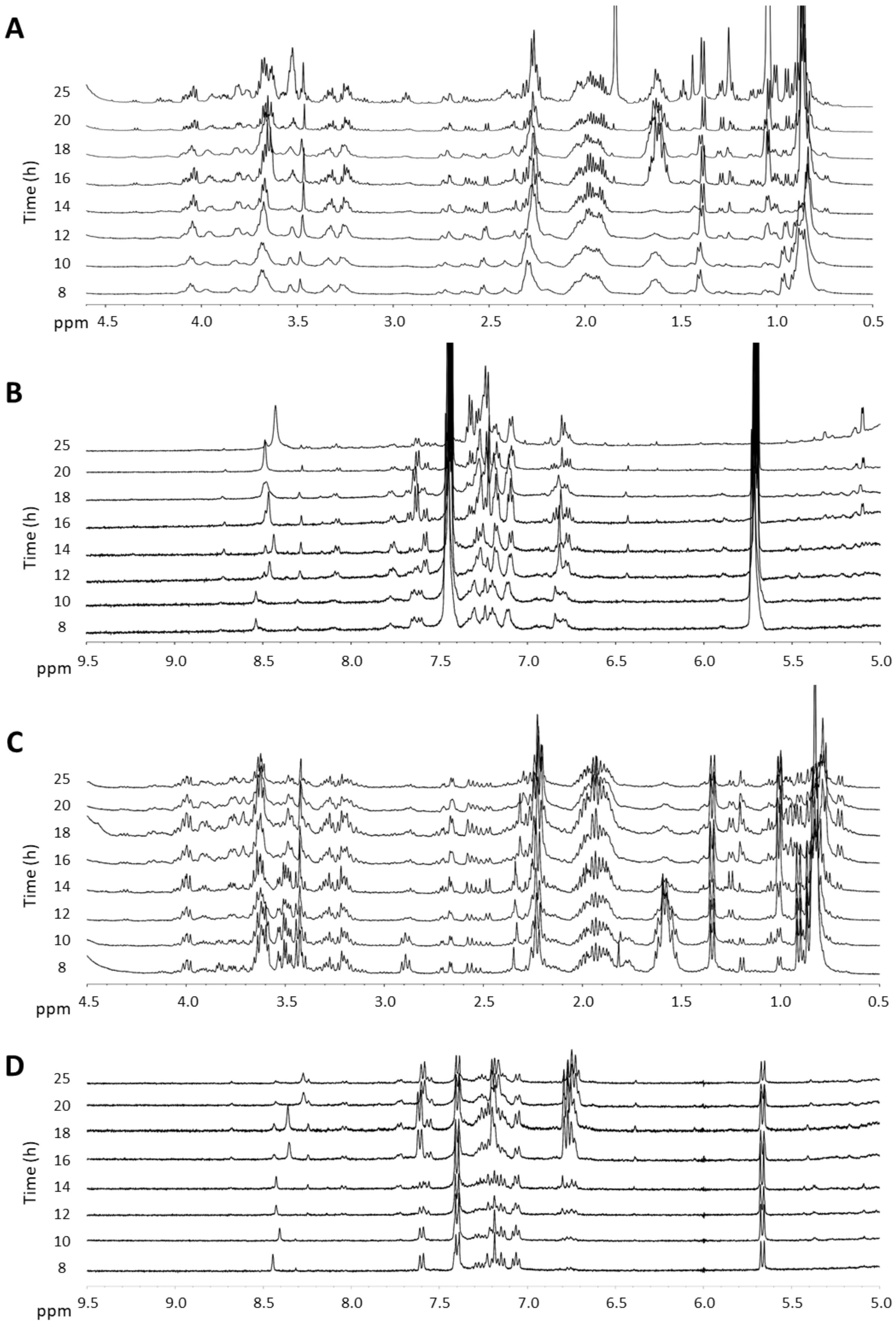

Figure S3. **Details of the ^{1}H NMR spectra in D_2O at different hours.** (A) EFC supernatant from 0.5 to 4.5 ppm; (B) EFC supernatant from 5.5 to 9.5 ppm; (C) LFC supernatant from 0.5 to 4.5 ppm; (D) LFC supernatant from 5.5 to 9.5 ppm.

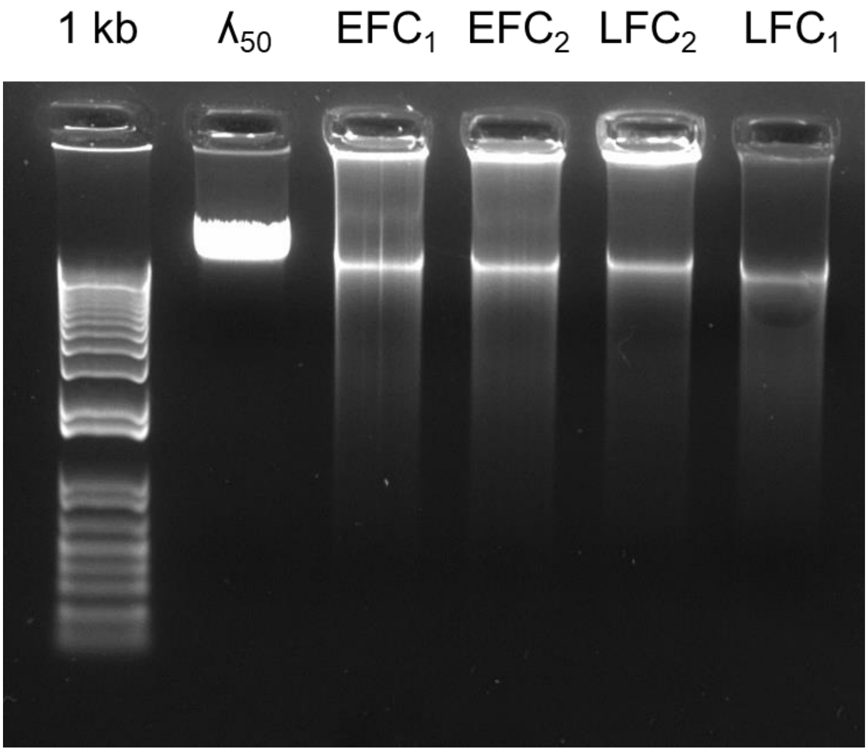

Figure S4. **Electrophoretic mobility in agarose gel (1% w/v) of the DNA obtained by direct REPLIg amplification of EFC and LFC supernatants**. Lane 1 from the left, 1Kb Plus DNA ladder; lane 2 lambda DNA; lane 3 and 4, DNA from EFC; lane 5 and 6, DNA from LFC.

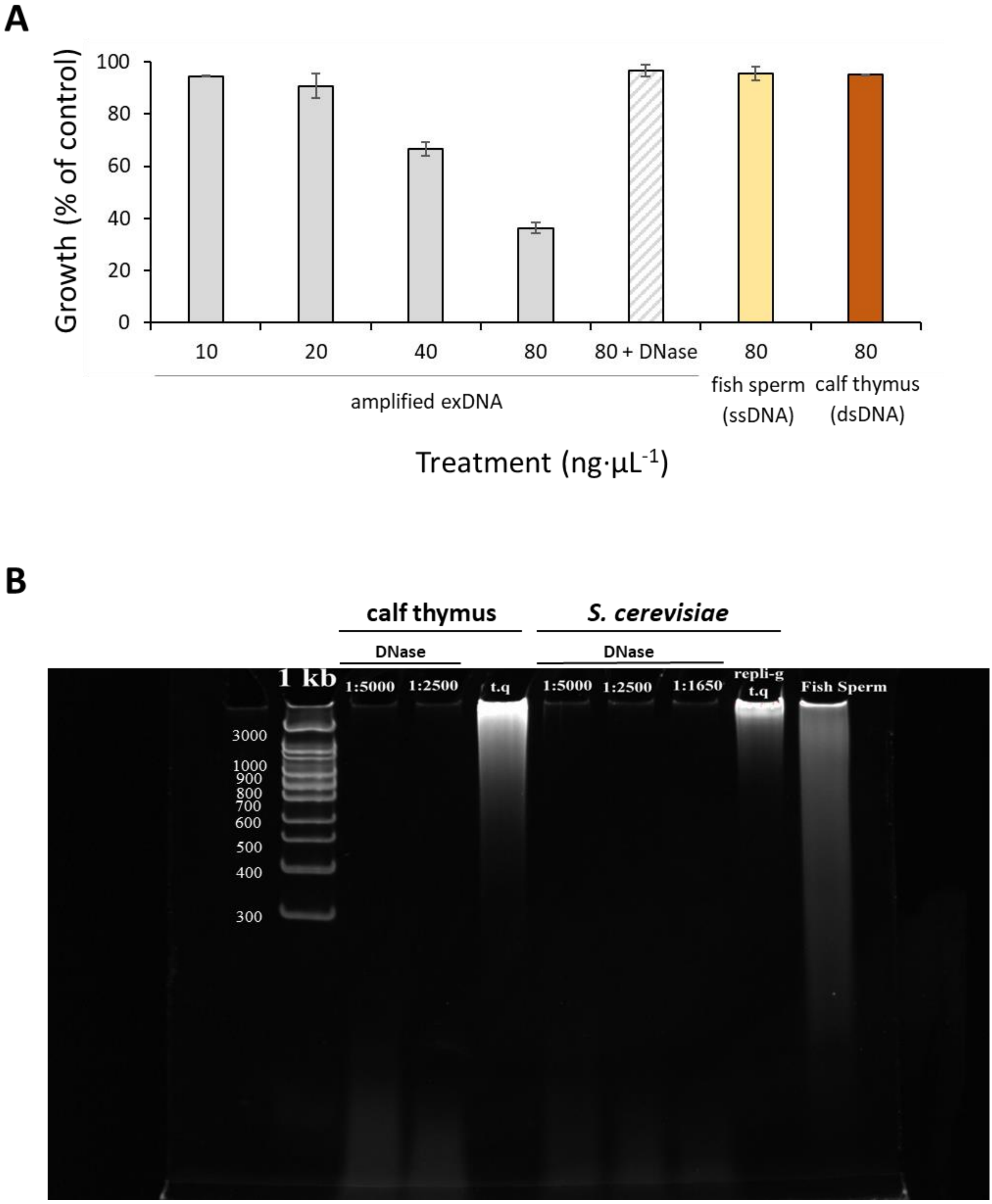

Figure S5. (A) Effect of different concentrations of DNA amplified from EFC supernatant on yeast growth in batch cultures. Null effects of heterologous DNAs from fish sperm and calf thymus are shown, as well as after pre-treatment of the amplified DNA with DNase. Data refer to means and standard deviations of 3 replicates. (B) Electrophoresis on 10% acrylamide gel of calf thymus DNA, DNA obtained by REPLIg amplification of EFC, and fish sperm DNA. DNase at different concentrations completely digested both calf thymus and EFC DNAs.

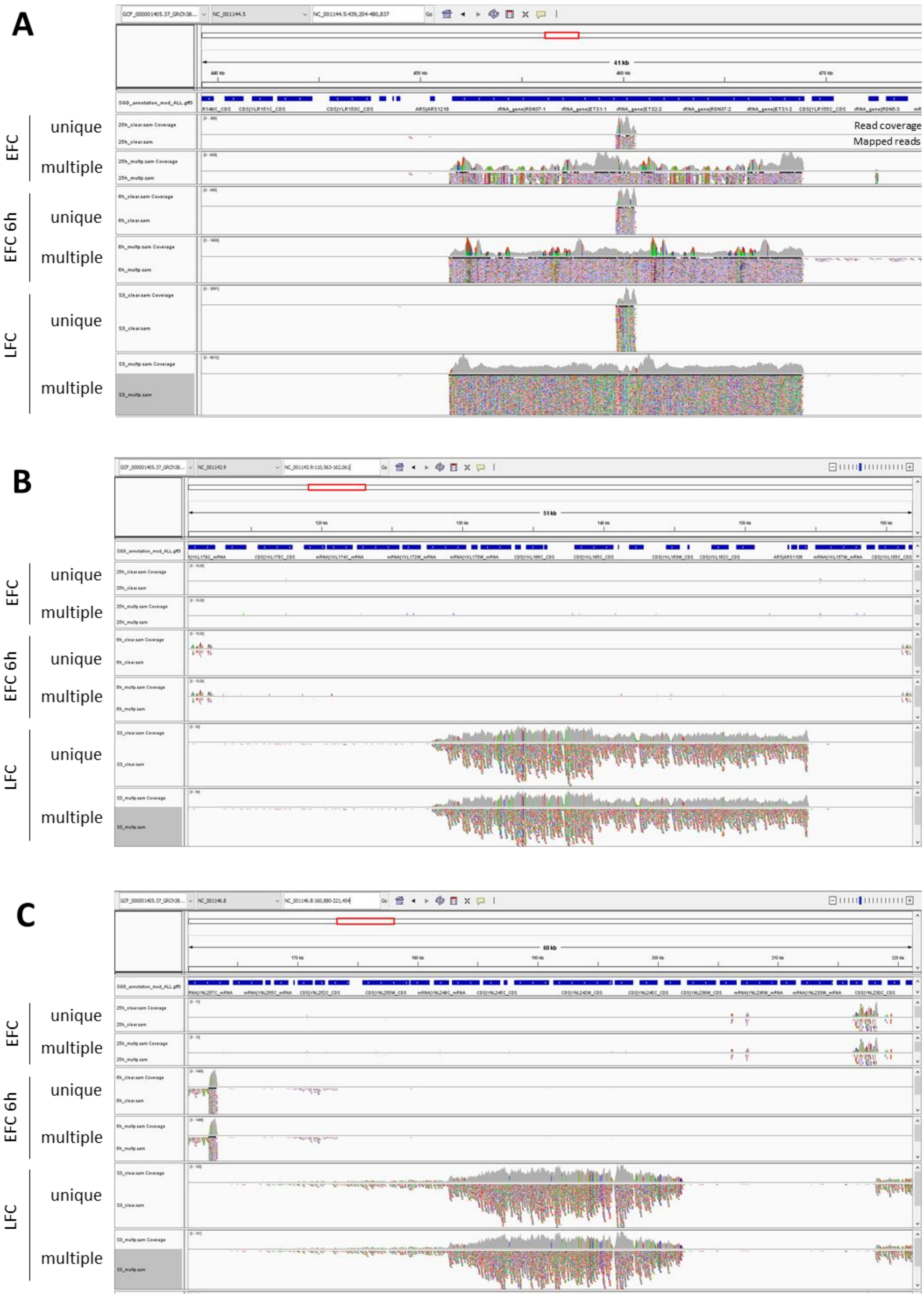

Figure S6 – Continued

D

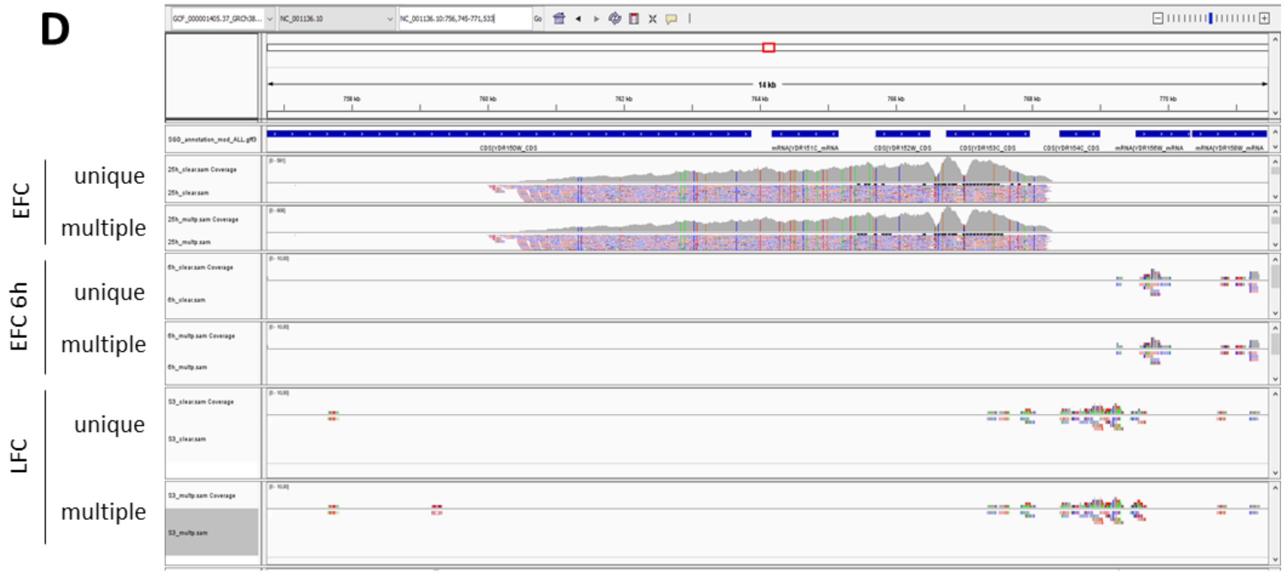

E

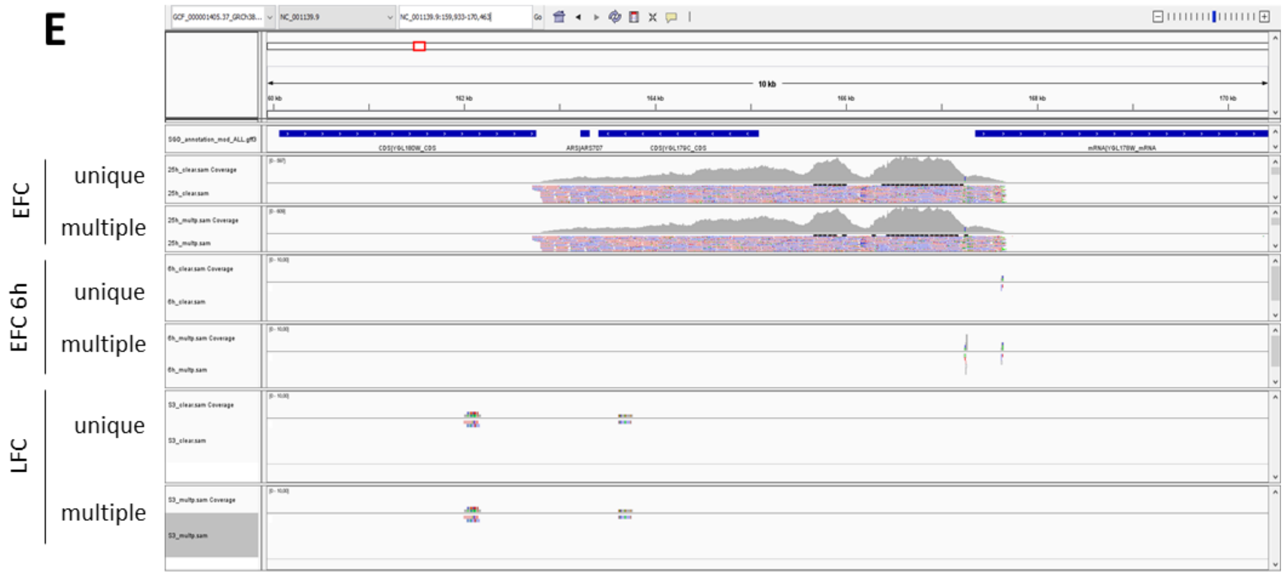

Figure S6. Examples of exDNA nucleotide reads mapped to the S288C *S. cerevisiae* reference genome using both "unique" and "multiple" approaches. Shown are samples of sequenced exDNA collected from media of fermentative EFC, early respiratory EFC-6h, and respiratory LFC. The reads are aligned on the corresponding parts of genomic regions of different chromosomes: (A) chromosome XII, (B) chromosome XI, (C) chromosome XIV, (D) chromosome IV and (E) chromosome VII.

A

Descriptions | Graphic Summary | Alignments | Taxonomy

Sequences producing significant alignments Download Select columns Show 100

select all *100 sequences selected* GenBank Graphics Distance tree of results MSA Viewer

Description	Scientific Name	Max Score	Total Score	Query Cover	E value	Per. Ident	Acc. Len	Accession
Saccharomyces cerevisiae strain CEN.PK113-7D chromosome XII	Saccharomyces ...	169	169	84%	1e-37	90.62%	1032974	CP046092.1
Saccharomyces cerevisiae strain ySR128 chromosome XII, complete sequence	Saccharomyces ...	169	169	84%	1e-37	90.62%	1076801	CP036478.1
Saccharomyces cerevisiae strain Y169 chromosome 12	Saccharomyces ...	169	169	84%	1e-37	90.62%	1061690	CP033481.1
Saccharomyces cerevisiae strain X55 chromosome 12	Saccharomyces ...	169	169	84%	1e-37	90.62%	1043571	CP033498.1
Saccharomyces cerevisiae strain SY14 chromosome I, complete sequence	Saccharomyces ...	169	845	84%	1e-37	90.62%	11848804	CP029160.1
Saccharomyces cerevisiae strain BY4742 chromosome XII, complete sequence	Saccharomyces ...	169	845	84%	1e-37	90.62%	1104511	CP026300.1
Saccharomyces cerevisiae strain CEN.PK113-7D chromosome XII, complete sequence	Saccharomyces ...	169	819	84%	1e-37	90.62%	1092391	CP022977.1
Saccharomyces cerevisiae strain HB_S_GIMBLETTROAD_14 chromosome XII sequence	Saccharomyces ...	169	169	84%	1e-37	90.62%	1077529	CP008264.1
Saccharomyces cerevisiae strain HB_C_TUKITUKI2_10 chromosome XII sequence	Saccharomyces ...	169	169	84%	1e-37	90.62%	1077494	CP008383.1
Saccharomyces cerevisiae strain T52 chromosome XII sequence	Saccharomyces ...	169	169	84%	1e-37	90.62%	1077515	CP008502.1
Saccharomyces cerevisiae strain HB_C_KOROKIPO_12 chromosome XII sequence	Saccharomyces ...	169	169	84%	1e-37	90.62%	1077771	CP008485.1
Saccharomyces cerevisiae strain HB_C_OMARUNUI_14 chromosome XII sequence	Saccharomyces ...	169	169	84%	1e-37	90.62%	1077859	CP008451.1
Saccharomyces cerevisiae strain HB_C_OMARUNUI_6 chromosome XII sequence	Saccharomyces ...	169	169	84%	1e-37	90.62%	1077614	CP008434.1
Saccharomyces cerevisiae strain WI_C_MBSP_4 chromosome XII sequence	Saccharomyces ...	169	169	84%	1e-37	90.62%	1077822	CP008519.1
Saccharomyces cerevisiae strain T16 chromosome XII, partial sequence	Saccharomyces ...	169	169	84%	1e-37	90.62%	1076869	CP008689.1
Saccharomyces cerevisiae strain MTKSKsf_E2 chromosome XII, partial sequence	Saccharomyces ...	169	169	84%	1e-37	90.62%	1077577	CP008128.1
Saccharomyces cerevisiae strain WSETAwf_B1 chromosome XII, partial sequence	Saccharomyces ...	169	169	84%	1e-37	90.62%	1077706	CP008094.1
Saccharomyces cerevisiae strain T8 chromosome XII, partial sequence	Saccharomyces ...	169	169	84%	1e-37	90.62%	1075190	CP008077.1
Saccharomyces cerevisiae strain HCNTHsf_C5 chromosome XII sequence	Saccharomyces ...	169	169	84%	1e-37	90.62%	1077600	CP007975.1
Saccharomyces cerevisiae strain HB_C_TUKITUKI2_4 chromosome XII, partial sequence	Saccharomyces ...	169	169	84%	1e-37	90.62%	1077518	CP007890.1
Saccharomyces cerevisiae strain ySR127 chromosome XII, complete sequence	Saccharomyces ...	169	169	84%	1e-37	90.62%	1062294	CP011558.1

B

Sequences producing significant alignments Download Select columns Show 100

select all *100 sequences selected* GenBank Graphics Distance tree of results MSA Viewer

Description	Scientific Name	Max Score	Total Score	Query Cover	E value	Per. Ident	Acc. Len	Accession
Saccharomyces cerevisiae strain HB_C_TUKITUKI1_16 chromosome XII sequence	Saccharomyces ...	150	150	75%	4e-32	93.88%	1077438	CP008400.1
Saccharomyces cerevisiae strain WSERCsf_G4 chromosome XII, partial sequence	Saccharomyces ...	150	150	75%	4e-32	93.88%	1077329	CP008213.1
Saccharomyces cerevisiae strain MARARsf_A10 chromosome XII, partial sequence	Saccharomyces ...	150	150	75%	4e-32	93.88%	1077957	CP008145.1
Saccharomyces cerevisiae strain HB_S_GIMBLETTROAD_9 chromosome XII, partial sequence	Saccharomyces ...	150	150	75%	4e-32	93.88%	1077536	CP008060.1
Saccharomyces cerevisiae strain CDRDR_sf_H chromosome XII sequence	Saccharomyces ...	150	150	73%	4e-32	94.74%	1077582	CP008009.1
Saccharomyces cerevisiae strain CEN.PK113-7D chromosome XII	Saccharomyces ...	148	148	75%	2e-31	93.88%	1032974	CP046092.1
Saccharomyces cerevisiae strain ySR128 chromosome XII, complete sequence	Saccharomyces ...	148	148	75%	2e-31	93.88%	1076801	CP036478.1
Saccharomyces cerevisiae strain Y169 chromosome 12	Saccharomyces ...	148	148	75%	2e-31	93.88%	1061690	CP033481.1
Saccharomyces cerevisiae strain X55 chromosome 12	Saccharomyces ...	148	148	75%	2e-31	93.88%	1043571	CP033498.1
Saccharomyces cerevisiae strain SY14 chromosome I, complete sequence	Saccharomyces ...	148	744	75%	2e-31	93.88%	11848804	CP029160.1
Saccharomyces cerevisiae strain BY4742 chromosome XII, complete sequence	Saccharomyces ...	148	744	75%	2e-31	93.88%	1104511	CP026300.1
Saccharomyces cerevisiae strain CEN.PK113-7D chromosome XII, complete sequence	Saccharomyces ...	148	722	75%	2e-31	93.88%	1092391	CP022977.1
Saccharomyces cerevisiae strain HB_S_GIMBLETTROAD_16 chromosome XII sequence	Saccharomyces ...	148	148	75%	2e-31	93.88%	1077615	CP008247.1
Saccharomyces cerevisiae strain WI_S_OAKURA_4 chromosome XII sequence	Saccharomyces ...	148	148	75%	2e-31	93.88%	1077541	CP008332.1
Saccharomyces cerevisiae strain T63 chromosome XII sequence	Saccharomyces ...	148	148	75%	2e-31	93.88%	1077176	CP008315.1
Saccharomyces cerevisiae strain HB_S_BILANCHER_12 chromosome XII sequence	Saccharomyces ...	148	148	75%	2e-31	93.88%	1077089	CP008298.1
Saccharomyces cerevisiae strain HB_S_BILANCHER_6 chromosome XII sequence	Saccharomyces ...	148	148	75%	2e-31	93.88%	1077803	CP008281.1
Saccharomyces cerevisiae strain HB_S_GIMBLETTROAD_14 chromosome XII sequence	Saccharomyces ...	148	148	75%	2e-31	93.88%	1077529	CP008264.1
Saccharomyces cerevisiae strain HB_S_GIMBLETTROAD_22 chromosome XII sequence	Saccharomyces ...	148	148	75%	2e-31	93.88%	1077481	CP008230.1
Saccharomyces cerevisiae strain HB_C_OMARUNUI_7 chromosome XII sequence	Saccharomyces ...	148	148	75%	2e-31	93.88%	1077484	CP008417.1
Saccharomyces cerevisiae strain HB_C_TUKITUKI2_10 chromosome XII sequence	Saccharomyces ...	148	148	75%	2e-31	93.88%	1077494	CP008383.1
Saccharomyces cerevisiae strain WI_S_JASA_13 chromosome XII sequence	Saccharomyces ...	148	148	75%	2e-31	93.88%	1077616	CP008366.1
Saccharomyces cerevisiae strain WI_S_JASA_5 chromosome XII sequence	Saccharomyces ...	148	148	75%	2e-31	93.88%	1076962	CP008349.1

C

Sequences producing significant alignments Download Select columns Show 100

select all *100 sequences selected* GenBank Graphics Distance tree of results MSA Viewer

Description	Scientific Name	Max Score	Total Score	Query Cover	E value	Per. Ident	Acc. Len	Accession
Saccharomyces cerevisiae strain CEN.PK113-7D chromosome XII, complete sequence	Saccharomyces ...	209	1012	100%	8e-50	92.05%	1092391	CP022977.1
Saccharomyces cerevisiae YJM451 chromosome XII sequence	Saccharomyces ...	209	15441	100%	8e-50	92.05%	1673802	CP006446.1
Saccharomyces cerevisiae YJM1615 chromosome XII sequence	Saccharomyces ...	209	21398	100%	8e-50	92.05%	2024659	CP006434.1
Saccharomyces cerevisiae YJM1208 chromosome XII sequence	Saccharomyces ...	209	31468	100%	8e-50	92.05%	2368220	CP006383.1
Saccharomyces cerevisiae YJM1202 chromosome XII sequence	Saccharomyces ...	209	18671	100%	8e-50	92.05%	1836195	CP006382.1
Saccharomyces cerevisiae YPH499 DNA, chromosome 12, complete sequence	Saccharomyces ...	209	3301	100%	8e-50	92.05%	1220201	AP026843.1
Saccharomyces cerevisiae strain S288C-SK1-cross isolate tetrad 8 spore a chromosome XII	Saccharomyces ...	209	2239	100%	8e-50	92.05%	1228789	CP089128.1
Saccharomyces cerevisiae PE-2 genome assembly, chromosome: XII	Saccharomyces ...	209	590	100%	8e-50	92.05%	1043062	HG994151.1
Saccharomyces cerevisiae PE-2 genome assembly, chromosome: XII	Saccharomyces ...	209	597	100%	8e-50	92.05%	1042334	LR999883.1
Saccharomyces cerevisiae strain CDRDR_sf_H chromosome XII sequence	Saccharomyces ...	207	207	100%	3e-49	91.45%	1077582	CP008009.1
Saccharomyces cerevisiae strain CEN.PK113-7D chromosome XII	Saccharomyces ...	206	206	100%	1e-48	91.45%	1032974	CP046092.1
Saccharomyces cerevisiae strain ySR128 chromosome XII, complete sequence	Saccharomyces ...	206	206	100%	1e-48	91.45%	1076801	CP036478.1
Saccharomyces cerevisiae strain Y169 chromosome 12	Saccharomyces ...	206	206	100%	1e-48	91.45%	1061690	CP033481.1
Saccharomyces cerevisiae strain X55 chromosome 12	Saccharomyces ...	206	206	100%	1e-48	91.45%	1043571	CP033498.1
Saccharomyces cerevisiae strain SY14 chromosome I, complete sequence	Saccharomyces ...	206	1030	100%	1e-48	91.45%	11848804	CP029160.1
Saccharomyces cerevisiae strain BY4742 chromosome XII, complete sequence	Saccharomyces ...	206	1030	100%	1e-48	91.45%	1104511	CP026300.1
Saccharomyces cerevisiae strain WI_S_OAKURA_4 chromosome XII sequence	Saccharomyces ...	206	206	100%	1e-48	91.45%	1077541	CP008332.1
Saccharomyces cerevisiae strain T63 chromosome XII sequence	Saccharomyces ...	206	206	100%	1e-48	91.45%	1077176	CP008315.1
Saccharomyces cerevisiae strain HB_S_BILANCHER_12 chromosome XII sequence	Saccharomyces ...	206	206	100%	1e-48	91.45%	1077089	CP008298.1
Saccharomyces cerevisiae strain HB_S_BILANCHER_6 chromosome XII sequence	Saccharomyces ...	206	206	100%	1e-48	91.45%	1077803	CP008281.1
Saccharomyces cerevisiae strain HB_S_GIMBLETTROAD_14 chromosome XII sequence	Saccharomyces ...	206	206	100%	1e-48	91.45%	1077529	CP008264.1
Saccharomyces cerevisiae strain HB_S_GIMBLETTROAD_22 chromosome XII sequence	Saccharomyces ...	206	206	100%	1e-48	91.45%	1077481	CP008230.1

Figure S7. BLAST based alignment of three different reads mapping on the rDNA region (chromosome XII) *versus* the GenBank nucleotide database. The three (A, B and C) most diverging sequences, i.e., those with the greatest number of mismatches, were selected.

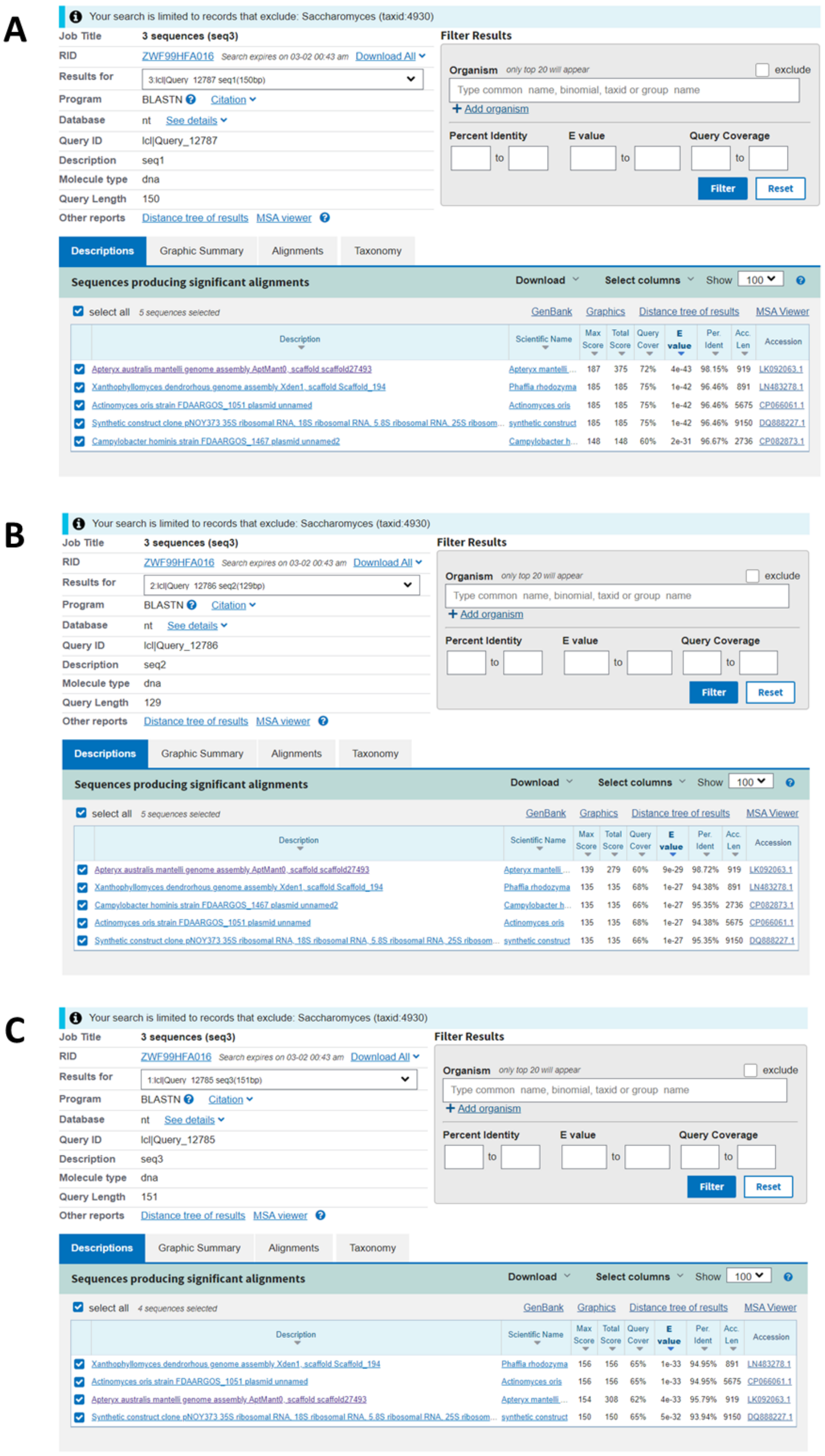

Figure S8. BLAST based alignment of three different reads mapping on the rDNA region (chromosome XII) *versus* the GenBank nucleotide database eliminating all possible hits matching versus "Saccharomyces" derived sequences, i.e., excluding all possible sequences referred to this term in the screened database. Results for the same three sequences reported in Figure S7 (A, B and C) are shown.

Table S1. **Results of two inhibition tests performed with *S. cerevisiae* CEN.PK1-2C treated with RNA extracted from either the same strain or human Hematopoietic Progenitor Cells (HPC).**

Inhibition test with RNA extracted from *S. cerevisiae* CEN.PK1-2C strain					
	**ng/uL**	**260/280**	**260/230**		
S. *cerevisiae* RNA	142.7	2.05	0.67		
S. *cerevisiae* RNA	150.2	1.98	0.73		
		**12 h**		**24 h**	
		**A_{590} (1:50)**	**A_{590}**	**A_{590} (1:100)**	**A_{590}**
**Control 1**		0.328	16.4	0.29	29.4
**Control 2**		0.355	17.75	0.29	28.7
**Treatment 1**	45 ng/uL S. *cerevisiae* RNA	0.345	17.25	0.31	30.9
	15 ng/uL S. *cerevisiae* RNA	0.337	16.85	0.32	31.9
	4,5 ng/uL S. *cerevisiae* RNA	0.354	17.7	0.3	30.2
**Treatment 2**	45 ng/uLi S. *cerevisiae* RNA	0.343	17.15	0.32	31.7
	15 ng/uL S. *cerevisiae* RNA	0.359	17.95	0.3	30.2
	4,5 ng/uL S. *cerevisiae* RNA	0.346	17.3	0.31	30.5

Inhibition test with RNA extracted from HPC cells					
	**ng/uL**	**260/280**	**260/230**		
HPC RNA	143	1.91	0.58		
HPC RNA	159.4	1.66	0.27		
		**12 h**		**24 h**	
		**A_{590} (1:50)**	**A_{590}**	**A_{590} (1:100)**	**A_{590}**
**Control 1**		0.328	16.4	0.29	29.4
**Control 2**		0.355	17.75	0.29	28.7
**Tratment 1**	45 ng/uL HPC RNA	0.327	16.35	0.27	26.9
	15 ng/uL HPC RNA	0.339	16.95	0.27	27.3
	4,5 ng/uL HPC RNA	0.355	17.75	0.28	27.7
**Treatment 2**	45 ng/uL HPC RNA	0.357	17.85	0.29	28.9
	15 ng/uL HPC RNA	0.353	17.65	0.29	28.8
	4,5 ng/uL HPC RNA	0.359	17.95	0.29	29

Table S2. List of reagents, yeast strains and software used in this work.

REAGENT or RESOURCE	SOURCE	IDENTIFIER
Chemicals, peptides, and recombinant proteins		
Deuterium oxide (99,8%)	Sigma-Aldrich	1.13366
DSS (Sodium trimethylsilylpropanesulfonate)	Sigma-Aldrich	613150
Potassium phosphate monobasic (KH_2PO_4)	Sigma-Aldrich	P8709
Sodium deuterium oxide (NaOD)	Sigma-Aldrich	372072
Glucose (dextrose)	SERVA Heidelberg	22720.01
D (+) Biotin	Sigma-Aldrich	851209
Ca D (+) pantotenate	Sigma-Aldrich	C8721
Nicotinic acid	Sigma-Aldrich	72309
Myo-inositol	Sigma-Aldrich	C5125
Thiamine hydrocloride	Sigma-Aldrich	T1270
Pyridoxal hydrocloride	Sigma-Aldrich	93759
para-aminobenzoic acid	Sigma-Aldrich	A9878
EDTA	Sigma-Aldrich	E5391
Manganese chloride tetrahydrate MnCl2 4H2O	Sigma-Aldrich	M3634
Cobalt(II) sulphate exahydrate CoCl2 6H2O	Sigma-Aldrich	C6768
Copper(II) sulphate pentahydrate CuSO4 5H2O	Sigma-Aldrich	C3036
Di-sodium molybdate dihydrate Na2MoO4 2H2O	Sigma-Aldrich	331058
Calcium chloride dihydrate CaCl2 2H2O	Sigma-Aldrich	223506
Iron sulphate-heptahydrate FeSO4 7H2O	Sigma-Aldrich	F8633
Boric acid H3BO3	Sigma-Aldrich	B6768
Potassium iodide KI	Sigma-Aldrich	221945
BD Bacto TM Casamino Acids	ThermoFisher Scientific	223050
Uracil	Sigma-Aldrich	U0750
Histidine	Sigma-Aldrich	H8125
Leucin	Sigma-Aldrich	L8000
Tryptophan	Sigma-Aldrich	T0254
Glutamic acid	Sigma-Aldrich	G1251
KOH	Sigma-Aldrich	P5958
NH4OH	Sigma-Aldrich	294993
Antifoam B	Sigma Aldrich	A5757
Yeast extract	TermoFisher Scientific	2127501
Bactopeptone	TermoFisher Scientific	211677
2’-Deoxyadenosine 5’-monophosphate	Sigma-Aldrich	D6375
2’-Deoxyguanosine 5’-monophosphate	Sigma-Aldrich	D9500
2’-Deoxycytidine 5’-monophosphate	Sigma-Aldrich	D7750
2’-Deoxythymidine 5’-monophosphate	Bio Basic	DB0367
ssDNA	Roche Diagnostics	11467140001
Critical commercial assays		
Ethanol-enzymatic kit from Megazyme	Megazyme International	K-ETOHLQR
SYTOX Green	Invitrogen	R37168
DNeasy® PowerMax® Soil Kit	Qiagen	47016
Hydroxyapatite DNA grade: Bio-Gel HTP	Bio-Rad	1300420
Qubit dsDNA assays Kits	Life Technology	Q32854
Qubit ssDNA assay kit	Life Technology	Q10212
Fish sperm ssDNA	Roche Diagnostics, Netherlands,	11467140001
DNase-free RNAse A	Sigma-Aldrich	11119915001
Deposited data		

DNA-Seq gene	This paper	Gene bank number
Experimental models: Organisms/strains		
Saccharomyces cerevisiae: Strain background: CEN.PK MATa; ura3-52 his3-D1 leu2-3,112 trp1-289 MAL2-8c SUC2	EUROSCARF (www.uni-frankfurt.de/fb15/mikro/euroscarf)	CEN.PK2-1C
Software and algorithms		
Kaluza Analysis Software v. 2.1	Beckman Coulter	N/A
^{1}H NMR v. 5.1.2	Mestrelab Research, Molfetta, Italy	www.inmr.net
Trim Galore package	http://www.bioinformatics.babraham.ac.uk/projects/trim_galore/	N/A
STAR software (version 2.4.2a)	Robinson et al, 2010	https://github.com/alexdobin/STAR/releases/tag/STAR_2.4.2a
Other		
Stericup® ®, 0,22 μm diameter	Millipore Express	32031623
